# Well‐being right before and after a permanent nursing home admission

**DOI:** 10.1002/hec.4595

**Published:** 2022-09-04

**Authors:** Judith Bom, Pieter Bakx, Sara Rellstab

**Affiliations:** ^1^ Erasmus School of Health Policy & Management Erasmus University Rotterdam Rotterdam the Netherlands; ^2^ Department of Economics Università della Svizzera Italiana Lugano Switzerland

**Keywords:** aging, anxiety and depression, control over one's life, loneliness, nursing home admission, well‐being

## Abstract

Permanent nursing home (NH) admissions are a frequent and major life event aimed at maintaining quality of life in old age. Yet, insights into the impact of a NH admission on well‐being are scarce and inconclusive. We evaluate the effect of a NH admission on domains of well‐being among those who are admitted using event study methodology for cross‐sections combined with inverse probability weighting. We apply this doubly robust approach to Dutch survey data on well‐being linked to extensive administrative data on NH admissions, health, and socio‐economic status. We find that a NH admission leads to a temporary increase in loneliness, the risk of anxiety and depression, and a loss of control over one's life. However, these scores revert to pre‐admission levels after 6 months. These findings may contribute to better‐informed individual‐level and policy decisions about potential NH entry and aging in place policies.

## 1 INTRODUCTION

A large share of older people spends their last years of life in a nursing home (NH). For example, the life‐time probability of being admitted to a NH is 56% for the US population aged 57–61 (Hurd et al., 2017) and 52% for the Dutch population aged 70 (Wouterse et al., 2021). The choice to permanently move to a NH is an important one. The motivation for moving to a NH (or for staying home) is often guided by concerns about the impact of the move on the resident's well‐being. However, insight in the well‐being of older people in these nursing homes is scarce and suffers from selection bias. This lack of evidence, combined with widespread apprehension about spending the last stage of life in a NH, means that heuristics and perceptions of the general public might not only affect the individual decision to move to a NH, but also policy measures regarding the financing and organization of NH care and substitute services such as home care and informal care. These heuristics may be shaped by what people know about nursing homes, but also by attributing problems that many older people face at the end of the life to the NH admission. Proper identification of the cause of problems may yield better‐informed decisions, at both the individual and the societal level.

In this study we provide insight into several domains of well‐being of older people in the months before and after a permanent NH admission. Thus, we seek empirical evidence that confirms—or contradicts—two conceptions about nursing homes and well‐being: (i) NH residents are worse off than the not‐yet‐admitted; (ii) NH residents “give up” on life once admitted.

To this end, we compare aspects of well‐being of comparable individuals who are either observed right before or after being admitted to a NH. Specifically, we estimate an event study model using Dutch survey data and extensive administrative data that is linked at the individual level for 10 cohorts of older people who were admitted to a NH permanently. By focusing on individuals who will enter or have entered a NH, we do not have to rely on a comparison with often highly different individuals who remain in the non‐institutionalized population. To explore and ensure the comparability of the survey respondents (about to be) admitted to a NH, we estimate the impact of a NH admission using a combination of inverse probability weighting based on a wide range of health, socio‐economic and demographic background information from administrative data and an event study method for repeated cross‐sections (Callaway & Sant’Anna, 2021).

A NH admission may be related to changes in well‐being in many ways. The admission itself may be highly stressful (Grenade & Boldy, 2008), and the move to a NH may for example, lead to a decrease in contact with friends and family members (Port et al., 2001). Moreover, a NH is a total institution (Goffman, 2009), which means that residents need to relinquish control of many aspects of their everyday lives and may thus experience—or at least perceive—a loss of control and of their identity. It may also lead to inactivity and passivity, as various tasks (e.g., cleaning, preparing meals) no longer have to be carried out. Dutch NH residents for example, tend to spend their days inactive (Ouden et al., 2015), while participation in activities could aid in maintaining physical health (Grönstedt et al., 2013) and well‐being (Buedo‐Guirado et al., 2020). This may mean that the health or well‐being of older people deteriorates faster after NH admission. However, NH admissions are also expected to have positive effects on dependent older people. Nursing homes provide individuals a safe environment, offering various types of care and social activities, which are valued by residents (van Campen & Verbeek‐Oudijk, 2017). Moreover, a NH facilitates being in contact with peers. Because these positive effects might in part cancel out with the negative effects, the relation between a NH admission and well‐being is an empirical question.

The decision to move to a NH permanently is even more important to study because it is a decision that is made under constraints, for example, regarding the information available about one's options and their impact on the outcomes. Moreover, the decision may be constrained by the availability of and eligiblity for public subsidies for nursing homes and substitute services such as home care and by constraints related to the amount of informal care that family members are able and willing to provide. Additionally, there may be time pressure. Hence, understanding the impact of the move is important.

The paper is related to two strands of the literature. First, it is related to studies on the association between a NH admission and elements related to well‐being like loneliness or experienced happiness (e.g., Böckerman et al., 2012; Kok et al., 2015; Olsen et al., 2016; Prieto‐Flores et al., 2011; Rapp et al., 2018; van Campen et al., 2018). The results in this literature are mixed, and they are likely to suffer from selection bias. As Young et al. (2017) point out based on a systematic literature review, these studies rely on comparisons between NH residents and older people living in the community. Since individuals with poor health and limited social networks are more likely to move to a NH, these results do not represent causal effects.

The second group of studies examines how a NH admission affects health care use and mortality outcomes. Kim and Lim (2015) use South Korean data to study the impact of subsidies for long‐term care (LTC) on informal care and medical care expenditures and find that for highly disabled individuals, substituting home care for NH care increases medical expenditures. Werner et al. (2019) estimate the differences in health care expenditures and mortality among US individuals who opt for either home care or NH care after a hospital discharge. In contrast to Kim and Lim (2015), they find that NH admissions lower hospital readmissions and expenditures, increase total health expenditures and have no impact on mortality. Lastly, Bakx, Wouterse, et al. (2020) find that being eligible for a NH admission in the Netherlands does not affect total health and LTC expenditures nor mortality but leads to a reduction in medical care expenditures.^1^

This paper contributes to the literature in two ways. First, we compare the well‐being of individuals about to be admitted to a NH to the well‐being of NH residents, and we make individuals comparable before NH admission with inverse probability weighting on detailed health, socio‐economic, and demographic dimensions using rich administrative data. This allows us to move closer to estimating causal effects of a NH admission on well‐being than the first strand of the literature listed above. Second, by focusing on aspects of well‐being, we examine a different outcome than previous studies on the effect of a NH admissions. As preserving quality of life is among the most important aims of LTC, this is a relevant outcome that has been understudied so far.

We find that a NH admission leads to a temporary increase in loneliness and in the risk of experiencing anxiety or depression, and to a loss of control over one's life. After 6 months, the scores on these aspects of mental well‐being restore to pre‐admission levels. So apart from a transitory adaptation period, these outcomes are not affected by a NH admission. These findings do not provide evidence that NH inhabitants are worse off than the not‐yet admitted. Our findings may be used for better‐informed decisions about a potential NH entry on the individual level. We do not find evidence for the premise underlying aging in place policies that the well‐being of older people is necessarily higher at home than in the NH. This may be because end‐of‐life problems are conflated with the effect of a NH entry: well‐being declines toward the end of life and this decrease coincides with a NH stay for many. We show that several well‐being related outcomes are already at a low level before entering a NH and therefore not caused by the NH entry. Hence, preserving the well‐being of older people might not be a good argument for further aging in place policies that encourage older people to postpone an admission, at least not for the aspects of well‐being this paper focused on and given the current Dutch eligibility rules.

## 2 NURSING HOME CARE IN THE NETHERLANDS

Nursing homes in the Netherlands provide around‐the‐clock support and care in an adapted and protective environment. Residents have the option to participate in recreative, social and cultural activities if they are offered (Zorginstituut Nederland, 2020).^2^ These activities can, for example, be sports, singing, crafting or playing games (Thuis in het Verpleeghuis, 2020), and may contribute to the well‐being of the residents. In addition to permanent NH care, there are two types of short‐term institutional care—post‐acute care and hospice care—but these are targeted at other, well‐defined groups of older people and are outside the scope of this study.^3^ The main alternative to NH care is home care, which may enable older people to live at home despite their limitations.

The use of publicly financed institutional care is rationed through an independent needs assessment. An independent agency assesses the eligibility for institutional care using centrally set, objective eligibility criteria (CIZ, 2018). Assessors decide which type and what volume of care one is entitled to receive. This care entitlement is expressed as a care package.^4^ There are few other barriers to access to LTC in the Netherlands because virtually all LTC—both home care and institutional care—is financed publicly through schemes that are universal and provide comprehensive coverage. The Netherlands is one of the largest public spenders on LTC worldwide; in 2019 total LTC accounted for 3.7% of the Dutch GDP (OECD, 2021). Public LTC insurance covers the costs of the care, the facilities, and room and board for NH residents. Co‐payments for institutional care cover only 8% of total expenditures (2019 level, Rijksoverheid, 2020), co‐payments for home care are either zero or a flat‐rate monthly fee of at most 17.50 euro (2019 level—Bakx, Bom, et al., 2020).

Nursing homes are contracted through regional single payers who each have a budget based on historical use. While this budget is binding, waiting lists are virtually absent during the study period in each of the regions (see e.g., CVZ, 2013). Still, elderly who are eligible for a NH admission may choose to wait until there is a place free in the specific NH that they may prefer, for example, because of its vicinity to where they live before the admission, and receive home care in the meanwhile.

Taken together, these features mean that an important subgroup of the Dutch elderly has a choice to move to a NH and decide about the timing of the move or to stay home with the help of home care (and informal care from family members and others). The public programs mean that both nursing homes and home care are affordable and accessible to everyone. Nursing home care is for people requiring intensive care and support, yet the extensive coverage of home care combined with few barriers to use, means there is an important subgroup of elderly for whom both types of arrangements are suited and available (Bakx, Wouterse, et al., 2020). The idea that many older people make a deliberate choice is reinforced by the observation that people generally do not move to a NH immediately after they become eligible, and some people who are eligible never move there at all (Bakx, Wouterse, et al., 2020; Tenand et al., 2020). Moreover, a sizable share of people recovers at home using home care after a hospitalization for a severe health problem such as a stroke or a hip fracture (Rellstab et al., 2020; Van den Burg et al., 2020). This finding shows that even people with a sudden increase in their need for LTC may choose to avoid a NH admission and use intensive home care instead.

## 3 DATA

### 3.1 Linked survey and administrative data

We use data from the Dutch Health Monitors of 2012 and 2016 about several outcomes related to health and well‐being of older people. The Health Monitor is a nationally representative survey conducted every 4 years starting from 2012 and consists of repeated cross‐sections of the 18+ population of the Netherlands. The surveys are self‐reported, there is no separate module for proxy‐interviews, but people may have received help with answering the questions from for example, friends, family members or care providers. When studying self‐reports in older populations, there may be a concern that not everybody is able to fill out a survey, and that therefore results may be biased. While this is a valid concern, this may apply to both older people in the NH and at home. Moreover, in our setting the problem is limited because our identification strategy ensures that the health status of all individuals is similar before their NH admission.

The data from the Health Monitor is linked at the individual level by Statistics Netherlands to Dutch administrative data using pseudonymized individual identifiers. We use administrative data about demographics (age, gender, marital status and household size), household income and wealth from the prior calendar year and, if applicable, the date of death of the respondent. Additionally, we link health‐related information on the type and duration of NH stays, the amount of home care use prior to the NH admission, type and duration of hospitalizations (by International Shortlist for Hospital Morbidity Tabulation (ISHMT) categories), use of pre‐admission prescription drugs^5^ (by Anatomical Therapeutic Chemical (ATC) classification code) and expenditures on care by general practitioner, hospital care, pharmaceuticals and total health care expenditures covered by mandatory social health insurance. A detailed overview of the used variables and datasets can be found in Supporting Information S1: Appendix 1.

For all individuals, we create a panel consisting of five observations covering a 6‐month time period, spanning from two time periods before admission up to three periods after admission.^6^ The panel is balanced for every variable except for the outcome variables from the survey, which are only observed once for every individual—in September 2012 or September 2016. As individuals are admitted to the NH at different points in time, we observe the outcomes for some respondents before NH admission, and for some after the NH admission.

### 3.2 Outcome measures

We use five outcome measures representing different aspects of well‐being that might be affected by a NH admission: loneliness, subdivided in social loneliness and emotional loneliness, the risk of experiencing depression and anxiety, and experienced inadequate control over one's life (only available in the 2016 survey). Supporting Information S1: Appendix 2 lists the exact definitions of these measures. While the measures do not capture overall well‐being, they are related to certain dimensions of quality of life (Eurostat, 2017), considered relevant by older people themselves (Hackert et al., 2019) and listed as outcomes in the Dutch national guideline for dementia care (Huijsman et al., 2020). Furthermore, they are likely to be affected directly by a “total institution” such as a NH (Goffman, 2009). Supporting Information S1: Appendix 3 depicts that these scores increase with age and in the years prior to death for the general Dutch population, reconfirming the relevance of these outcomes for the elderly population.

### 3.3 Sample selection

We restrict the sample to respondents aged 75 and over for whom information regarding at least one of the well‐being measures is available. To observe well‐being in the months before or after the NH admission in the Health Monitor, we focus on respondents who were interviewed in 2012 and admitted to a NH between March 2011 and September 2013 (referred to as the 2012 respondents) or interviewed in 2016 and admitted to a NH between March 2015 and September 2017 (the 2016 respondents).

We only include individuals whose NH stay lasted at least 365 days because this solves two issues. First, this restriction ensures that the respondents observed in the period after NH admission are still residing in a NH when answering the survey. Second, by restricting our sample to long‐stay NH admissions we make the earlier groups (individuals observed before admission) more comparable to the later ones (individuals already residing in a NH for several months). Individuals who will only make use of NH care for a short period because of either very good or very poor health are excluded from the not‐yet‐admitted groups using this selection criteria. The restriction also implies that individuals who die within 1 year after admission, about 30% of the admitted individuals in our study period, are not considered. This implies that the results are only valid for people who have a low probability of dying within a year. The reasons for and implications of this restriction are discussed in more detail in the methods section.

## 4 METHODS

We use an event study framework for cross‐sections to compare well‐being of respondents who are interviewed up to 1 year before a NH admission to respondents who have lived in a NH for up to 1.5 years (Callaway & Sant’Anna, 2021). Our approach consists of two main steps. First, we take advantage of the administrative data to make groups of respondents that are admitted to a NH in five different periods of time comparable in health, socio‐economic and demographic characteristics one period before their own NH admission with inverse probability weighting. Second, we use an event study model to determine the impact of a NH admission on several domains of well‐being among these comparable individuals who have been admitted to a NH at different calendar times, and hence have filled out the survey on health and well‐being measures in different event times.

To group respondents in our analysis, we rely on two different time dimensions: (1) calendar time *T*_*k*_, or the *kth* 6 month period away from the survey^7^; and (2) event time *s*_*q*_, or the *q*th 6 month period away from a NH admission. Both calendar and event time are measured in 6‐month intervals. We assign the survey respondents into five groups^8^ based on the time since or until NH admission when answering the survey.^9^

Since we do not observe the self‐reported outcomes over time, one may be concerned that individuals admitted to a NH in the beginning of the time span may have different well‐being levels before being admitted to a NH than individuals admitted at the very end. To address this concern, we use the extensive information available in the administrative data summarized in a propensity score that is aimed at making all individuals comparable one period before their respective NH admissions. To be able to use all relevant variables in the propensity score model and to make the groups comparable at admission on as many dimensions as possible, we estimate the propensity scores for the entire Dutch population that was admitted to a NH for at least 365 consecutive days in the period when the survey respondents we analyze were admitted to a NH (between T_‐3_ and T_1_).

For this population, we estimate the propensity of being admitted to a NH in the period when the first group, group 5, is admitted to a NH, in calendar time *T*_−3_. Hence, we estimate the probability of belonging to group 5 using a logit model for both the 2012 and the 2016 survey separately (Equation (1)):^10^ ^:^

(1) $$P\left(NH_{T_{-3},g}\right)=\Lambda \left(X_{T_{-4},g}\beta_{g}+\varepsilon_{g}\right),\;for\;g\in \left\{2012,\;2016\right\}$$

The control variables *X* contain information about health status and care need, availability of NH care substitutes, and socioeconomic status from the administrative data one period before the admission of the first group, *T*_‐4_. Supporting Information S1: Appendix 5 lists all variables that are included in *X*. Thus, the propensity score model captures the probability of being admitted to a NH at the same time as group 5, given a complete set of time‐variant determinants of NH care use (de Meijer et al., 2009, 2013)—the individual's health status and care need, availability of care substitutes and socioeconomic status—in the 6 months preceding the admission of the first admitted group.

Subsequently, we use the coefficients of this propensity score model to predict the probability of being admitted *in event time s*_0_, the period of the own NH admission, for the subsample that answered to the Health Monitor surveys (Equation (2)) using information from the 6 months prior to their actual NH admission (*s*_−1_).

(2) $$\hat{P}\left(NH_{s_{0},g}\right)=\hat{p}\left(X_{s_{-1},g}\right)=\Lambda \left(X_{s_{-1},g}\hat{\beta_{g}}\right),\;\mathrm{for}\;g\in \left\{2012,\;2016\right\}$$

As the propensity score model is based on predictors from administrative data, we observe all the inputs into the propensity score models at different points in calendar and event time. This allows us to predict the propensity score for all individuals based on information 6 months prior to their actual NH admission (instead of their characteristics before group 5 was admitted in *T*_−3_), implying that we make groups comparable one period before their own admissions. This procedure corrects for any underlying health, socio‐economic or demographic differences between the groups 1–5 at the same event time (instead of calendar time). At the same time, applying $\hat{\beta_{g}}$, estimated when predicting the probability of being admitted in the first group, in the prediction of the propensity scores in (2) ensures that any policy changes between the admission of group 5 and 1 that would change the coefficients in Equation (1) do not influence the estimates as we apply the policy regime of *T*_−3_ to all groups.^11^

In the final step, we use the propensity scores in a doubly robust approach: we regress the well‐being scores $Y_{i,T_{0}}$ on time away from the NH admission *s*_*q*_ while including control variables (Equation (3)) and applying the inverse probability weights using the estimated propensity scores from Equation (2).

(3) $$Y_{i,T_{0}}=\alpha +\sum_{q=-2}^{2}\delta_{q}s_{q}+\gamma_{1}age_{i,s_{0}}+\gamma_{2}male_{i}+\gamma_{3}married_{i,T_{0}}+\gamma_{4}med{ication}_{i,s_{-1}}+\gamma_{5}eli{gibility}_{i,s_{0}}+\gamma_{6}hos{pitalization}_{i,s_{0}}+\nu_{i,T_{0}}$$

We control for the following variables that may be correlated with both time away from NH admission and well‐being: age at admission, gender, being married at the time of the survey *T*_0_, having been hospitalized at admission *s*_0_, the type of NH care that the person is eligible for at admission *s*_0_, and, to proxy for chronic health problems, indicators for taking antithrombotics (ATC B01), diabetes related drugs (ATC A10), drugs for obstructive airway diseases (ATC R03), and medication to treat acid‐related disorders (ATC A02)^12^ in the 6 months prior to admission *s*_−1_.^13^ In this step, we pool the 2012 and 2016 survey samples, as both samples are highly comparable in terms of pre‐admission characteristics after weighting.

### 4.1 Identifying assumptions

To understand to what extent coefficients δ_*q*_ from Equation (3) measure the causal effect of a NH admission and what are the threats to this interpretation, we discuss five identifying assumptions (Callaway & Sant’Anna, 2021).^14^

**A1: Irreversibility of the treatment.** This assumption implies that once an individual is admitted, the individual will continue to live in the NH. The intuition behind this assumption is that otherwise, the impact of the admission may be conflated with the impact of the NH discharge or differences in composition between the groups because of selective mortality. In our analysis, we ensure that this assumption is satisfied by limiting our sample to people who are (about to be) admitted for a permanent NH stay rather than short stays in nursing homes for rehabilitative purposes. That is, we only select people whose stays last at least 1 year. This restriction, which here means having a balanced panel in the panel event study case, and is commonly imposed, comes at an obvious cost: it means that we are presenting results for the selected group of older people who are about to move to a NH and who do not die until a year after. Therefore, the relationship we find in this sample may not be generalized to those for whom it is highly unlikely to ever move to a NH, for example, because of their preferences or their health status; or for the very sick who have a very limited life expectancy at the time of admission. In a robustness test, we limit the study sample further by excluding all long‐term admissions that started for rehabilitation purposes^15^ and by focusing on those respondents staying for at least 180 days.

**A2: No compositional changes over time.** This assumption applies to the case of repeated cross‐sections only. It ensures that observed changes in well‐being are driven by time away from the NH admission rather than group composition. The differences in observable characteristics between the groups at admission are small in the unweighted sample, and even smaller after the propensity score weighting (Table 1 & Supporting Information S1: Appendix 7). While the sample selection and the inverse probability weighting do not fully rule out that there may be differences in unobservables, we believe that the set of observable characteristics in the administrative data covers all relevant domains. Nursing home care use is determined by a combination of characteristics related to health, functional limitations, demographics, socio‐economic status, the availability of alternatives (de Meijer et al., 2009, 2013) and all these are covered.^16^ It is unlikely that our results are biased by the lower ability to fill out a survey in a NH due to health problems. The propensity score weighting makes sure that all cohorts are in comparable health status before their admission, thereby eliminating this potential source of bias. Moreover, we have arguably reduced differences in preferences considerably by only selecting people who end up being admitted within the same 2.5 year time frame.

**A3: Limited treatment anticipation.** This assumption implies that respondents do not have complete control over when they are admitted to the NH. Most individuals in our sample are probably aware of the approaching NH admission and the fact that it is likely permanent. Indeed, many of them have applied for NH eligibility. However, while people in our sample do anticipate a NH admission, it is unlikely that they are able to anticipate—let alone determine—the exact timing of the admission. In the Netherlands, eligibility for NH care does not imply immediate admission, because of a combination of demand‐side factors and supply‐side factors. For instance, while waiting lists are virtually non‐existent at the regional level (e.g., CVZ, 2013), a place in the preferred NH may not be available right away. There is quite some variation in the time between the eligibility decision and the admission within groups with similar health, limitations, and other characteristics (Bakx, Wouterse, et al., 2020; Tenand et al., 2020).^17^

**A4: Conditional parallel trends based on not‐yet treated groups.** The intuition behind this assumption is that individuals in each of the groups are on similar well‐being trajectories in the periods before the admission. In most event study frameworks, evidence for the common trend assumption is provided by showing that pre‐trends are zero. Since the parallel trend assumption relies on non‐realized potential outcomes, this procedure is no formal evidence for parallel trends but rather an indication that the assumption is plausible. With cross‐sectional data and no control group, it is even more difficult to provide evidence about the plausibility of this assumption than with panel data, as we only observe outcomes of all groups once. Hence, we cannot show levels or trends in outcomes before the NH admission for all groups. The usual test of zero pre‐trends is therefore less informative with cross‐sectional data, as it only implies that the level in well‐being of group 1 in period *s*_−2_ is similar to the level of well‐being of group 2 in period *s*_−1_, but it does not convey any information on whether the groups have parallel trends in outcomes before the treatment. Conversely, a non‐zero pre‐trend may still be compatible with A4 if all groups experience the same non‐zero pre‐trend in the (unobserved) outcome. Instead, we take advantage of the rich administrative data to show trends in care use before NH admission, which are likely indicative of underlying trends in related domains of health and well‐being. Related, following the work of Freyaldenhoven et al. (2021), we study the potential role of confounding on the interpretation of our estimates by extrapolating the trend from the pre‐event trend and thus evaluate the robustness of the effect estimates to assuming no trend (main analysis) versus a continuing trend.

**A5: Common** **support** **for propensity scores.** To ensure that the groups are comparable, we exclude people whose propensity score is outside the common support. That is, we exclude 120 observations from the 2012 sample and 61 observations from the 2016 sample for whom the probability of an admission is so high or so low that there are no comparable people in the other groups.

## 5 RESULTS

### 5.1 The propensity of a long‐stay nursing home admission

Being eligible for LTC is the strongest predictor for a long‐stay NH admission, according to the propensity score models (Supporting Information S1: Appendix 6, Table A6.1). Additionally, higher age and intensity of home care receipt predict an admission. After excluding the observations outside the common support of the propensity scores, our final sample consists of 2255 respondents. For these observations, the propensity score distributions of the different groups largely overlap (Supporting Information S1: Appendix 6, Figure A6.1). The overlap implies that—within this population and given the control variables—it is “as good as randomized” who enters the nursing in the first 6 month period and who enters later.

### 5.2 Descriptive statistics, stability of group composition and parallel trends

The propensity scores are used to make our sample of health‐survey respondents comparable to each other. Table 1 presents a subset of pre‐admission characteristics for all five groups after weighting our data. The full balancing tables before and after weighting of our data can be found in Supporting Information S1: Appendix 7. Descriptive statistics reveal that the study sample is old (85 years on average) and that the large majority is female and living alone in the months preceding the NH admission. Moreover, they are in poor health and frail: a large share of them had an inpatient hospitalization in the 6‐month period prior to the NH admission and the majority uses medication to treat a chronic illness.

**TABLE 1:** Balancing table pooled sample before nursing home admission (weighted)

Health monitor 2012 + 2016, weighted	Group 1	Group 2	Group 3	Group 4	Group 5
	Mean	Mean	Mean	Mean	Mean
Age in *s*_0_	85.9	85.6	85.6	85.2	84.9
Male	0.33	0.32	0.30	0.27	0.33
Living with partner in *s*_−1_	0.32	0.35	0.38	0.34	0.29
Eligible for care packages 1–4 in *s*_0_	0.33	0.35	0.32	0.32	0.35
Eligible for care packages 5 & 7 in *s*_0_	0.39	0.39	0.29	0.35	0.29
Eligible for care packages 6 & 8 in *s*_0_	0.24	0.17	0.21	0.14	0.20
Eligible for care package 9 in *s*_0_	0.04^a^	0.09	0.18	0.20	0.16
Hospitalization in *s*_0_	0.39	0.43	0.51	0.38	0.34
Antithrombotics in *s*_−1_	0.58	0.57	0.54	0.58	0.58
Drugs for acid‐related disorders in *s*_−1_	0.52	0.51	0.56	0.52	0.57
Drugs for obstructive airway diseases in *s*_−1_	0.18	0.15	0.18	0.19	0.11
Drugs for diabetes in *s*_−1_	0.20	0.19	0.25	0.26	0.26
Observations	1048	729	187	168	123

*Note*: Differences between groups are calculated using standardized differences between group 5 and one of the other groups.

^a^ standardized difference >0.25 following the threshold of Stuart et al. (2013). Event time *s*_0_ refers to the period of the nursing home admission, and *s*_−1_to the 6 month period before nursing home admission. We distinguish between four types of nursing home care eligibility grouping similar care packages. The care packages are explained in Footnote 4.

The five groups are largely comparable in demographics, health, and care need. The only meaningful difference detected is that Group 1 is less likely to be eligible for NH care from care package 9 than Group 5.^18^ We test the robustness of our results to excluding this group in one of our robustness checks. This high comparability between the groups is evidence that we are likely to satisfy assumption 2, no compositional changes across groups.

**FIGURE 1:**
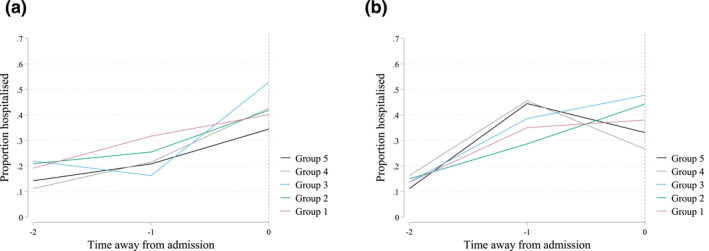
Proportion of individuals within cohort making use of hospital care within 6 month intervals before nursing home (NH) admission. (a) 2012 sample. (b) 2016 sample

To inspect the plausibility of assumption 4, conditional parallel trends of the not‐yet‐treated, we plot for all groups pre‐admission trends of health care use which are indicative of how respondents' health changed during this period. Figure 1 shows the pre‐admission average shares of hospitalization for each of the groups. Before NH admission, the groups follow similar trends in terms of hospitalizations. Pre‐admission trends concerning home care use and health care expenditures are reported in Supporting Information S1: Appendix 8. These analyses indicate that all groups follow similar trends in terms of health care expenditures. Moreover, for the 2012 Health Monitor sample, the proportion of home care users before admission is roughly similar among the five groups of respondents, although average home care hours slightly increase for every subsequent group.^19^ Finding parallel trends in health care use before NH admissions provides evidence for the assumption that the groups follow similar health and well‐being trajectories.

### 5.3 Raw differences in domains of well‐being before and after nursing home admission

**FIGURE 2:**
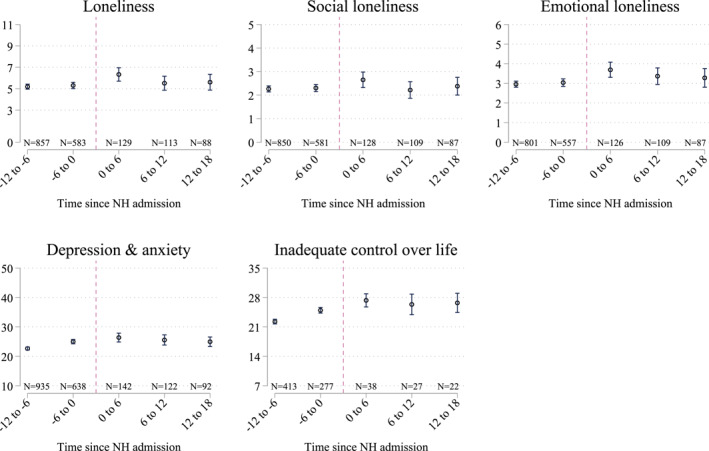
Outcomes by time since nursing home (NH) entry (2012 & 2016 pooled). Bars indicate 95% confidence intervals. Results are weighted such that the cohorts are comparable at NH admission. The underlying values are presented in Supporting Information S1: Appendix 9. The outcome inadequate control over life is only reported for the 2016 sample

To explore how NH entry is associated with different domains of well‐being, we plot the weighted scores for the respondents in Figure 2. These scores indicate that many respondents are dealing with well‐being issues both before and after admission. Following Van Tilburg and de Jong Gierveld (1999), we consider individuals lonely when they score at least a three on the (emotional or social) loneliness scale. The group of individuals considered lonely can be further split into a group experiencing moderate loneliness (score 3–8) and a group with severe loneliness (9–11). The average loneliness score is above five in all groups, suggesting many of the observed individuals are experiencing loneliness, both before and after NH admission. These scores are much higher than the scores observed in the general elderly population, where only the average loneliness‐score of individuals aged 95 and above is higher than 5 (Supporting Information S1: Appendix 3). Moreover, respondents on average score above the cut‐off of >22 indicating inadequate control over one's life (Pearlin & Schooler, 1978; RIVM, 2021b). For the depression and anxiety scores, cut‐offs are defined at >15 for moderate risk and >30 for high risk (Kessler et al., 2002; RIVM, 2021a). The average respondent in our sample falls within the range considered of experiencing a moderate risk. Again, these scores are substantially higher than the average scores observed in the general elderly population (Supporting Information S1: Appendix 3).

Visually inspecting the differences in scores across the groups, we observe higher scores among the group that is just admitted to a NH compared to the group interviewed in the months before the admission. However, for respondents who have been in the NH for a longer time, scores gradually restore to the pre‐admission levels, except for inadequate control of one's life.

### 5.4 Doubly robust approach

**FIGURE 3:**
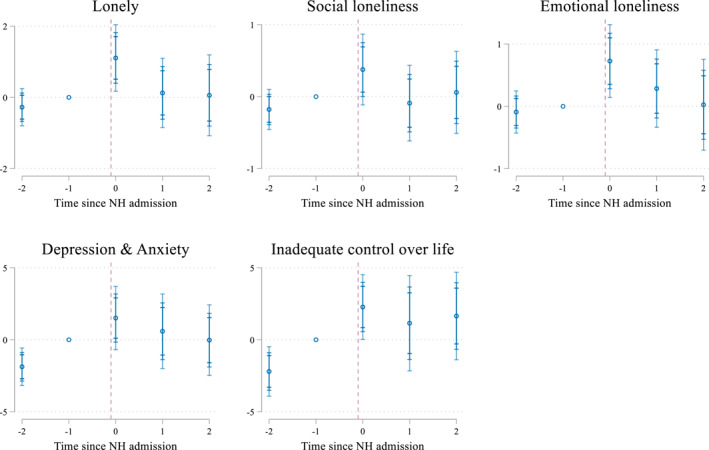
Effect of a nursing home (NH) admission on aspects of mental well‐being (pooled sample). Estimates of $\hat{\delta_{q}}$ and their 90, 95, and 99 confidence intervals. Underlying estimates are presented in Supporting Information S1: Appendix 10. The outcome inadequate control over life is only reported for the 2016 sample

Figure 3 presents $\hat{\delta_{q}}$, the coefficients for the indicator for the time relative to the NH admission for the pooled sample. The regression analyses confirm the results from the descriptive analysis. Before the NH admission, we find stable scores for the different measures of loneliness. In contrast, the negative coefficients for period *s*_−2_ (compared to the reference category at event time *s*_−1_), suggest a small increase in the risk of anxiety and depression and an increased loss of control in the cohorts leading up to the admission. While these scores represent deteriorating well‐being before the NH admission, they do not necessarily invalidate assumption three of limited treatment anticipation. Experiencing increasing levels of anxiety and loss of control at home does not automatically mean that one can anticipate the exact timing of the admission or know whether the move will be permanent. Additionally, as discussed in the methods‐section, observing pre‐trends does not necessarily invalidate assumption four of conditional parallel trends as long as all groups follow a similar trajectory.

The observed deteriorations in the estimates during the pre‐treatment period do however suggest that if we would observe a further decline, this could not be ascribed to the NH admission. However, if anything, the results for the post‐admission periods show that loss of control and the increase in anxiety and depression, do not continue in the same trend as before, indicating that the deterioration in the outcomes stops around 6 months after the NH admission.

Well‐being deteriorates (represented by increased scores) in the 6 months after NH admission (event time *s*_0_) and restore in the periods afterward (event times *s*_1_‐*s*_2_). Loneliness increases by 1.1 points (total scale) in the period right after admission, which is the sum of a 0.4 point increase in social loneliness and a 0.7 point increase for emotional loneliness. While this increase does not push the average individual over the threshold of experiencing severe loneliness, the increase is not negligible. Individuals experiencing spousal bereavement for example, report increases in emotional loneliness scores of 1–2 points (Szabó et al., 2020). In the periods more than 6 months after admission, individuals in our sample however seem able to recover from the increase in loneliness as scores restore to baseline levels.

We draw similar conclusions when considering experienced control over life and risk of anxiety and depression. The increase of 1.5 points pushes the average respondent slightly further above the threshold of inadequate control, but on average this threshold was already passed before the admission. This increase is slightly larger in size than the increase of about one point that has been found after experiencing any negative life event (like increased arguments with your partner or someone in the family experiencing a major financial crisis) at old age (Cairney & Krause, 2008).

Similarly, the increase of 2.3 points in the risk of anxiety and depression does not push the average individual into the high‐risk category but can still be considered relevant. For example, individuals with risk for anxiety & depression scores ranging between 20 and 24 show to consult their doctor for mental health problems on average 1.9 times compared to 3.4 times for individuals scoring 25–29 (Andrews & Slade, 2001).

## 6 ROBUSTNESS CHECKS

### 6.1 Robustness to data and modeling choices

**FIGURE 4:**
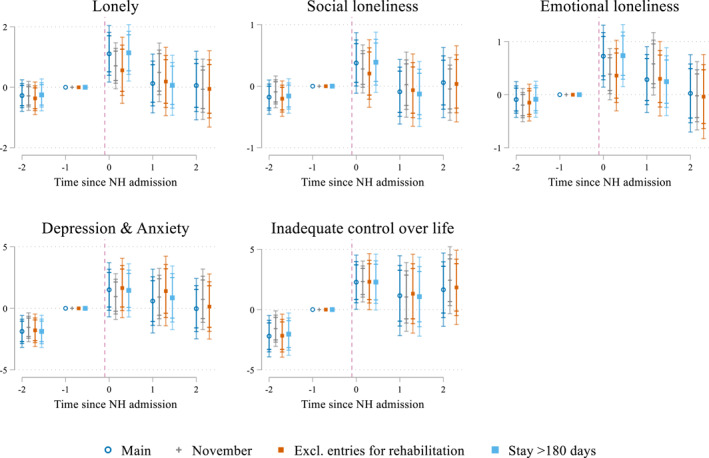
Robustness checks (pooled sample). Estimates of $\hat{\delta_{q}}$ and their 90, 95, and 99 confidence intervals. The following results are presented: “Main” corresponding to the main results using the pooled sample, “November” when shifting the interview date from September 1^st^ to November 1^st^, “Excl. entries for rehabilitation” pooled sample where individuals who entered the nursing home (NH) for rehabilitative purposes (care package 9) are excluded and “Stay >180 days” the pooled sample where all NH admissions lasting at least 180 days are kept. Underlying estimates are presented in Supporting Information S1: Appendix 11. The outcome inadequate control over life is only reported for the 2016 sample

To test the robustness of our results to data and modeling choices, we have performed several checks. Figure 4 presents the results. First, we explore how the uncertainty regarding the exact moment that the survey was filled out affects the results by shifting the interview date from September 1 (the first date that the survey was sent out) to November 1. This means that we change the assignment of the respondents to the five groups based on the timing of the survey relative to their interview. The results suggest that the uncertainty about the exact date the survey was filled out does not affect the interpretation of the results.

Second, we exclude all individuals who entered a NH for rehabilitative stays (care package 9) to evaluate whether a stricter definition of a permanent NH stay affects our results. The results reveal that excluding this group, which is rather small, does not affect the results.

Third, we run our analyses when considering NH stays of at least 180 days instead of our restriction to 365 days. Most individuals now included in this shorter‐stay sample are individuals who did not live for a full year in the NH because they passed away. For this analysis we hence exclude individuals observed when already living for at least 12 months in a NH (Group 5), as we cannot ensure comparability of this group with the others anymore. The results of this shorter‐stay sample look comparable to the main outcomes.

Lastly, in Supporting Information S1: Appendix 11 we present estimates (1) when running the analyses separately for the 2012 and 2016 sample and (2) when using logit models (with binary outcomes) instead of the ordinary least squares models used in the main analyses. For both checks the models yield results that are comparable to the results from the main analyses.

### 6.2 Potential confounding

The doubly robust procedure mitigates the differences between the five cohorts at the time of the admission through the inverse probability weighting and through adding controls to the event study regression. In this subsection, we further explore the potential role of gradual changes, for example, in health status, in the period around the NH admission. For this, we build on the argument made by Freyaldenhoven et al. (2021) that a pre‐trend is a sign of confounding. We study this in two steps. First, we extend the length of the pre‐trend to four periods (i.e., 2 years) and use this longer pre‐trend to study the potential role of confounding on the interpretation of our estimates by extrapolation of the pre‐trend. Second, we explicitly explore how physical health, which is a potential confounder and not controlled for in the event study regression because it may be endogenous to well‐being, evolves over time. We only discuss the results of these analyses here, Supporting Information S1: Appendix 12 contains a more detailed motivation and explanation.

**FIGURE 5:**
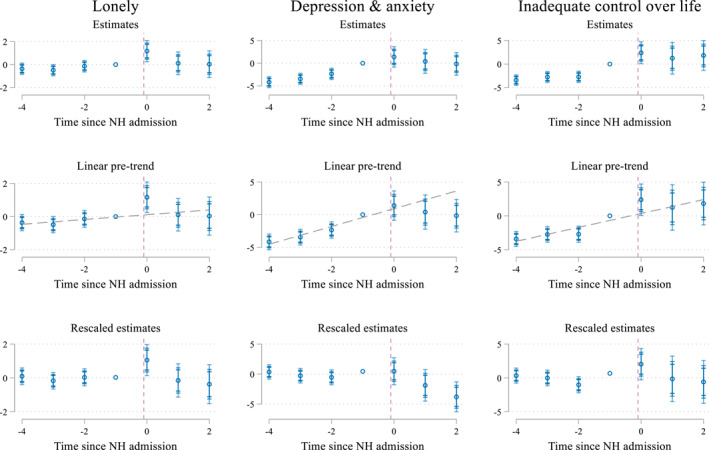
Effect of a nursing home (NH) admission on aspects of mental well‐being (pooled sample) while (lower panel) rescaling estimates to indicate deviations from the trend line. Top panel: Estimates of $\hat{\delta_{q}}$ and their 90, 95, and 99 confidence intervals. Middle panel: Estimates of $\hat{\delta_{q}}$ and their 90, 95, and 99 confidence intervals with a fitted linear pre‐trend. Bottom panel: rescaled estimates as deviations from the trend line estimated in the middle panel. Separate graphs for emotional and social loneliness are available in Supporting Information S1: Figure A12.2 in the Appendix. The outcome inadequate control over life is only reported for the 2016 sample

Figure 5 displays the results when extending the number of pre‐event periods to four 6 month periods (top panel) and extrapolating the trend in the pre‐treatment period to model the distortion of the event study estimates caused by potential confounding (mid panel).^20^ The bottom panel rescales the estimates to show deviations from the trend line instead of differences compared to period *s*_−1_ (Doyle et al., 2018; Freyaldenhoven et al., 2021). The graphs show that loneliness estimates also return to pre‐admission levels when calculating the deviation from the pre‐admission trend. Put differently, these figures suggest that the main results for the loneliness‐related outcomes presented in Figure 3 are robust to the effect of the confounders causing the pre‐trend, which was very limited for loneliness. Moreover, the results, confirm that perceived control over life was already on the decline, but stabilized after a NH admission. For anxiety and depression, the analyses even indicate that, if there is a change at all, a NH admission decreases the risk of anxiety and depression. This reconfirms that the main findings for loss of control are robust to changing the assumption of no confounding, while the effect on the risk for anxiety and depression is more sensitive which assumption is made about confounding.

**FIGURE 6:**
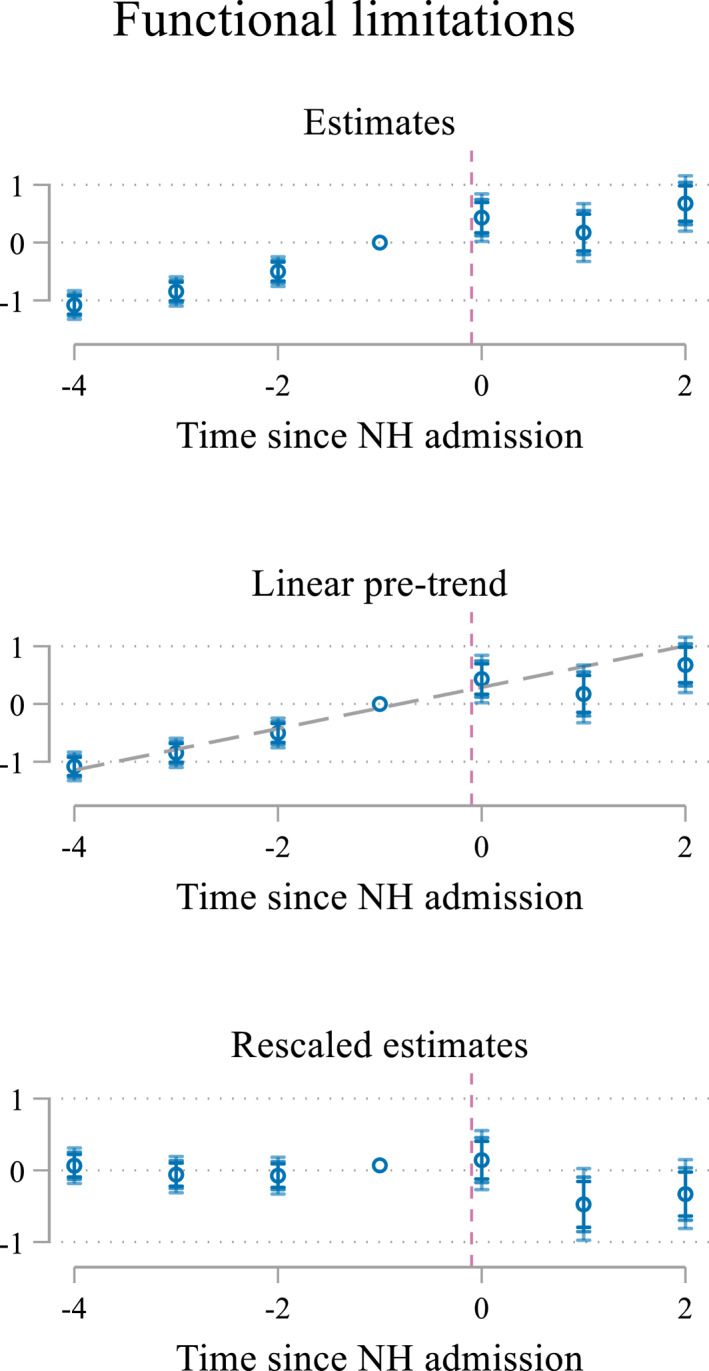
Effect of a nursing home (NH) admission on functional limitations (pooled sample) while (lower panel) rescaling estimates to indicate deviations from the trend. Top panel: Estimates of $\hat{\delta_{q}}$ and their 90, 95, and 99 confidence intervals. Middle panel: Estimates of $\hat{\delta_{q}}$ and their 90, 95, and 99 confidence intervals with a fitted linear pre‐trend. Bottom panel: rescaled estimates as deviations from the trend line estimated in the middle panel. Functional limitations are measured through a 7 item list of tasks performed everyday life: Listening to a conversation with three or more persons; having a conversation with one person; reading the small print of a newspaper; recognizing someone's face at a distance of 4 m; carrying an item of 5 kg for 10 m; reaching for something on the ground; walking for 400 m without standing still

Figure 6 depicts the results when using functional limitations as outcome measure. The top panel confirms that the number of functional limitations grows both before and after a NH admission. This reconfirms that our identification strategy only makes individuals comparable right before admission (at event time *s*_−1_), but it does not remove the expected physical health decline over time. Additionally, the deviations from a linear increase in functional limitations are very close to zero in the four periods before a NH admission. This implies that imposing a linear trend on the well‐being estimates, as done in Figure 5, may work well to account for the potential confounding role of functional limitations. Furthermore, this finding indicates that the well‐being estimates are not simply the result of changes in functional limitations: if that were the case, the well‐being scores would have followed a similar trend as the one observed for the number of limitations, both before and after the NH admission. While the outcomes, possibly except for loss of control, follow a linear increasing trend before the admission that is similar to the linear trend in functional limitations; the trend is not continued after admission. Hence, we interpret these results as suggestive evidence that the role of functional limitations as a confounder is limited.

## 7 DISCUSSION AND CONCLUSION

A NH admission is a major life event and preventing NH admissions is an important policy goal in many countries. Moreover, nursing homes are generally aimed at preserving the well‐being of residents despite their functional limitations. Perceptions about quality of life in a NH by the general population may have a large effect on private decisions and on public policy, for example, about LTC financing. Hence, understanding the relationship between a NH admission and well‐being of older people is crucial for improving NH policy. Yet, this evidence is thus far limited.

This paper compares outcomes on several measures related to well‐being of people just before NH entry to those just after a permanent NH entry. We use the Dutch Health Monitor combined with Dutch administrative data on NH admissions and background characteristics to identify the timing of the survey interview compared to the NH admission. We make survey respondents comparable at the time of their NH admission following the approach proposed by Callaway and Sant’Anna (2021), thereby eliminating selection bias arising in studies that compare all NH residents to all older people in the community.

We find no changes in loneliness before the NH admission and only transient differences among respondents who have recently been admitted to a NH compared to those who have not yet been admitted. Furthermore, we find that the perceived control over one's life and feelings of anxiety or depression were already on a decline. This decline continues in the period right after the admission but stabilizes thereafter. Based on our study we cannot infer whether this halt can be ascribed to adaptation, the new living environment, or changes in medical treatment at the NH. Together, these findings, however, mean that we do not find evidence that nursing homes have a large negative effect on one's well‐being or that longer‐term NH residents “give up” after the admission.

This study uses unique linked survey and administrative data from the Netherlands. When considering how the results can be generalized to other settings, two institutional characteristics may matter. First, the group for which a NH admission is a relevant alternative as well as the timing of such an admission may be different across countries. Access to NH care in the Netherlands is equitable for everyone because of the combination of comprehensive, universal public coverage, income‐dependent co‐payments, objective needs‐based eligibility criteria, and sufficient supply. At the same time, extensive public subsidies for home care mean that people with functional limitations may postpone an admission longer than if such subsidies were not available. Second, the NH characteristics may shape the influence a NH has on the well‐being of residents. In the Netherlands, NH quality is arguably fairly uniform because the access and provider payments are not linked to the income or wealth of residents meaning that rich residents get the same services as others.

Our findings mean that there is no evidence for permanent changes in aspects related to well‐being due to a NH admission. Although this might be counterintuitive, a potential explanation for this is that many attribute end‐of‐life problems that older people face to the NH admission. Many older people in a NH report high levels of loneliness and anxiety and low levels of control over one's life. However, these scores are similar among individuals who will be admitted to a NH soon. Thus, the NH admission does not seem to be an important reason for these low levels of reported well‐being. Our findings may contribute to better‐informed decisions about a potential NH entry at the individual level.

At the societal level, the findings may be used to inform policy decisions, regarding aging in place and how the budget is allocated between NH residents and frail older people living at home. First, the descriptives presented in the paper show that the oldest old not only face physical and cognitive problems, but that their well‐being is also poor, as many report loneliness, inadequate control over life and they are more often at risk for anxiety and depression. Hence, public policy should also be targeted at preventing and reducing these problems rather than at physical and cognitive problems alone.

Furthermore, the Netherlands and other countries encourage older people to live at home as long as they can and support them intensively through aging in place policies. One of the premises of these policies is that the well‐being of older people is higher at home than in the NH. While there may be other good reasons to facilitate aging in place, this study shows that protecting the well‐being of the oldest old may not be one of them (at least not for the outcomes considered in this study).

Finally, the Dutch government has in recent years increased LTC spending by 10% to improve the well‐being of NH residents (Rijksoverheid, 2017). While this study shows that well‐being issues are indeed common in this population, we also find that on average the loneliness, risk of anxiety and depression and control over one's life of older people living in the community is equally poor. Hence the well‐being of older people living at home warrants as much attention as the well‐being of nursing homes residents.

## CONFLICT OF INTEREST

None of the authors has a conflict of interest.

## Supporting information

Supporting Information S1

## Data Availability

This research uses non‐public data available at Statistics Netherlands.

## ENDNOTES

^2^
Municipalities organize and finance similar activities for individuals living at home.

^3^
Furthermore, in the past there used to be a distinction between residential care homes and nursing homes, where residential care homes catered to older people with less severe limitations than nursing homes. This distinction disappeared after 2010. As the eligibility criteria became stricter many residential care homes closed or merged with nursing homes (Verbeek‐Oudijk & Van Campen, 2017). In the study period, nursing homes accounted for the vast majority of the admissions.

^4^
There are 10 different care packages: (1) sheltered living environment with support, (2) sheltered living environment with support and care, (3) sheltered living environment with support and comprehensive care, (4) sheltered living environment with intensive support and comprehensive care, (5) protected living environment with intensive dementia care, (7) protected living environment with very intensive care, because of specific health conditions, with an emphasis on support, (6) protected living environment with intensive personal care and nursing, (8) protected living environment with very intensive care, because of specific health conditions, with an emphasis on personal care and nursing, (9) rehabilitative care and (10) terminal palliative care. Care package nine is only registered in our dataset in the beginning of the study period due to changes in the way this care type is financed.

^5^
We use the information prior to admission since the data on prescription drugs is only available for out‐patient prescriptions.

^6^
We use calendar year information in case information is not available on a daily level (i.e., for information from the Tax Office, health insurance and medication use).

^7^
Invitations for the questionnaires were sent out by the Community Health Services in the fall of the interview year, not earlier than September 1^st^. Data was gathered between September and November in 2012 and September and December in 2016 (GGD, 2012; GGD‐GHOR, 2016). We use 1 September as the reference date when calculating the time between the nursing home admission and finishing the survey.

^8^
We opt for five groups as a compromise between data points and observations: as few individuals are observed after nursing home admission, we are unable to focus on smaller time frames (e.g., 3 months periods).

^9^
An explanation of the construction of the different groups based on the timing of the survey and the nursing home admission can be found in Supporting Information S1: Appendix 4.

^10^
We estimate separate propensity scores for the 2012 and 2016 samples, as a long‐term care reform in 2015 may have changed the availability of home care, among other things; and because the reform changed the way the data is recorded.

^11^
For example, if a person had to have a greater need for care to be eligible for a nursing home in 2013 than in 2011 and we compare well‐being after admission for both groups, the well‐being of the 2011 group may be higher because they are on average in better health. The inverse probability weights will correct for this policy‐induced well‐being difference by giving more weight to people who are admitted in 2013 who are similar to the people who were admitted in 2011.

^12^
These are among the most used prescription drugs among Dutch older people and are for treatment of chronic illnesses. Hence, people who use one of these drugs have usually used them over a longer period.

^13^
Since the survey sample is relatively small, we do not include all variables from the propensity score estimation in the main analysis.

^14^
Note that the numbering of assumptions does not coincide entirely with the numbering in Callaway and Sant’Anna (2021), as their assumptions cover more than one possible empirical set‐up.

^15^
Our preferred specification is to only restrict on length of stay, as there may also be other nursing home residents whose purpose of stay is not rehabilitative but who expect to stay in the nursing home only for a very short period.

^16^
Time‐invariant characteristics such as preferences regarding home care versus nursing home care are taken care of by the selection of the study population, who eventually all move to a nursing home.

^17^
Furthermore, the group of nursing home residents who were admitted for post‐acute rehabilitative care may not have anticipated the admission as much as the rest of the sample. In addition, they may not have anticipated that the admission ended up being permanent. While this group is too small to study it separately, we have excluded it from the sample in a robustness check that is presented in Section 6.1.

^18^
This difference can be explained by a minor change in the long‐term care system for the small group of people eligible for care package 9.

^19^
We do not report home care trends for the 2016 sample. In 2015 the organization of home care (and hence the data collection) changed as a result of a LTC reform, which limits the comparability of home care data in different pre‐admission years for this subsample.

^20^
The linear trends fit the trend in the pre‐event periods entirely for all outcomes, except for the outcome loss of control in event time *s*_−2_, where the coefficient is still borderline significant at 5%. For this reason, we also fit a quadratic trend as an alternative robustness check in Supporting Information S1: Appendix Figure A12.1. However, the quadratic trend is more likely to distort the rescaled coefficients in event time *s*_1_ and *s*_2_, and hence we prefer to present the more conservative results here using the linear fit.
