# Supplementary material for: Well‐being right before and after a permanent nursing home admission
Source: Health Econ. 2022 Sep 4;31(12):2558–74. doi: 10.1002/hec.4595 (PMC9826495; doi:10.1002/hec.4595)
Supplement: Supplementary file 1 — Supporting Information S1 [file HEC-31-2558-s001.docx]

**Appendix 1 - Overview used variables and datasets**

**A1.1 Survey data**

We use data from the Dutch Health Monitors of 2012 and 2016 about the mental well-being of older people. The Health Monitor is a nationally representative survey conducted every four years starting from 2012 and consists of repeated cross-sections of the 18+ population of the Netherlands. Individuals were invited by a letter to respond to an online survey. Depending on the region, a paper questionnaire was either sent along with the initial request or sent later in case of nonresponse. Surveys via telephone or in person were rare: 0.5% in 2012 and 0.1% in 2016 (CBS, 2015, 2017). In 2012, about 700.000 individuals were invited to participate to any of the Health Monitors, 387.195 individuals responded to the questionnaire. In 2016, about 460.000 of the roughly 1.15 million approached individuals responded. Separate versions of the survey either target the total Dutch population, youth, or older people. As we focus on older people, we limit our sample to the data collected by the Community Health Services via the survey specifically aimed at the 65+ population.

Table A1.1: Overview of the administrative data that are used in this article

| **Variable** | **Measurement** | **Time** | **Source data, CBS code** |
| --- | --- | --- | --- |
| Age | Classified into age groups:  -75-80 years old  80-85 years old  -85-90 years old  -90+ years old  Highest category used in category changed within time period. | Per 6-month period | Municipal register, GBAPERSOONTAB |
| Gender | 1=Male | - | Municipal register, GBAPERSOONTAB |
| Living together with partner | 1=Yes (Unmarried couple; Married couple; Couple with children)  Latest status used in case status changed within time period. | Per 6-month period | Municipal register, GBAHUISHOUDENS2018BUSV1 |
| Living alone | 1=Yes (Alone living)  Latest status used in case status changed within time period. | Per 6-month period | Municipal register, GBAHUISHOUDENS2018BUSV1 |
| Widowed | 1= (Partner at t-1, alone in t0)  Latest status used in case status changed within time period. | Per 6-month period | Municipal register, GBAHUISHOUDENS2018BUSV1 |
| Married | 1=Married or registered partnership  Latest status used in case status changed within time period. | Per 6-month period | VERBINTENISPARTNERBUS |
| Foreign descent | 0=No  1=Yes, 1^st^ or 2^nd^ generation | - | Municipal register, GBAPERSOONTAB |
| Household income | In quintiles | Per calendar year | Tax records, INHATAB |
| Household wealth | In quintiles | Per calendar year | Tax records, VEHTAB |
| Home care & Home care hours | -Any  - Total number of hours of homecare received | Per 6-month period | LTC Administration Office (CAK), GEBZZVTAB |
| Spending on nursing + personal care | Total value of use * tariff for personal or nursing home received at home | Per 6-month period | LTC Administration Office (CAK), GEBZZVTAB |
| Hospitalization  & Length of Stay Hospitalization | - Any - Total length of stay for all hospital admissions within 6 month period | Per 6-month period | Dutch Hospital data, LMR_BASIS |
| Diagnosis hospitalization | By ISHTM (in case of hospitalization) | Per 6-month period | Dutch Hospital data, LMR_BASIS |
| Drug use | - Any - By ATC | Per 6-month period | Zorginstituut, MEDICIJNTAB |
| Health care expenditures | - Total expenditures - Expenditures GP - Expenditure hospital - Expenditures pharmaceuticals | Per calendar year | VEKTIS, ZVWZORGKOSTENTAB |
| Short-term nursing home admission | Admitted for <365 days | Per 6-month period | LTC Administration Office (CIZ), INDICAWBZTAB |
| Length of stay short term nursing home admission | Total number of days in nursing home during this 6 month period | Per 6-month period | LTC Administration Office (CIZ), INDICAWBZTAB |
| Long-term nursing home admission | In case admitted for at least 365 days | Per 6-month period | LTC Administration Office (CIZ), INDICAWBZTAB |
| Type of nursing home admission | Highest care package score within 6 month period | Per 6-month period | LTC Administration Office (CIZ), INDICAWBZTAB |
| Type of nursing home admission at admission | Care package score assigned closest to admission | - | LTC Administration Office (CIZ), INDICAWBZTAB |
| Dementia | 1 if individual received indication for nursing home admission based on psychogeriatric problems | Per 6-month period | LTC Administration Office (CIZ), INDICAWBZTAB |

**Appendix 2 - Definition of well-being measures**

Table A2: Definition of well-being measures

| **Well-being measure** | **Definition** |
| --- | --- |
| Loneliness | Loneliness is assessed based on the 11 questions of the De Jong Gierveld scale (1999) which defines loneliness as the discrepancy between one's desired and achieved levels of social relations. A distinction is made between the following types of loneliness:   - Social loneliness: the absence of an acceptable social network, a wider circle of friends and acquaintances that can provide a sense of belonging, of companionship and of being a member of a community. - Emotional loneliness: the absence of an attachment figure in one's life and someone to turn to.   The scores range from:   - Loneliness: (0) no to (11) severe loneliness - Social loneliness: (0) no to (5) severe social loneliness - Emotional loneliness: (0) no to (6) severe emotional loneliness |
| Risk of feeling depressed or anxious | Respondents were asked 10 questions related to feelings of depression and anxiety in the past 4 weeks, based on the Kessler Psychological Distress Scale (Kessler et al., 2002). Answers are transferred into a score between 10-50. A score of 10-15 represents no or a low risk; 16-29 moderate risk and 30-50 high risk of anxiety or depression. (RIVM, 2021a) |
| Inadequate control over one’s life | Respondents were asked 7 questions related to control over one’s life following the Pearlin & Schooler Mastery Scale (1978). Answers are transferred into a score between 7-35. For ease of interpretation the scores are reversed, meaning that now a high score indicates little control. A score of 7-22 represents average to much control; 23-35 indicates inadequate control (RIVM, 2021b) |

**Appendix 3 - Well-being and physical health trends**

To gain insight into the health and well-being of the entire population, figure A3.1 plots scores of several measures by age group for all 65+ respondents of the Health Monitors of 2012 and 2016. In line with what we would expect, they show that health and well-being deteriorate with age. Especially the number of functional limitations measured using the OECD-scale increases with age. Social loneliness has a rather flat curve, with only a slight increase with age. Figure A3.2 shows health and well-being trends in terms of time away from death. Here slopes are less steep, health and well-being scores are already relatively high in the years preceding death. Again, however, all indicators for having health problems and well-being issues increase with death approaching, except for social loneliness, which remains stable. These descriptive statistics suggest that these measures are good proxies of the dimensions of health and quality of life that matter for this age group.

Figure A3.1: Health and well-being by age group (N=390.270)

**

Note: Respondents from Health Monitor 2012 and 2016 combined, except for the outcome inadequate control over life is only reported for the 2016 sample.

Figure A3.2: Health and well-being five years before death (N=46.208)

Note: Respondents from Health Monitor 2012 and 2016 combined, except for the outcome inadequate control over life is only reported for the 2016 sample.

**Appendix 4 – Explanation of the construction of the different groups based on the timing of the survey and the nursing home admission**

Figure A4.1 provides a visual representation of the construction of the different groups based on the timing of the survey and the nursing home admission, and it shows how calendar time and event time relate in our set-up for the example of the 2012 survey. The same structure applies for the 2016 survey. The classification of respondents into groups is as follows: Group 1 contains respondents who were interviewed 6-12 months before their nursing home admission ($in event time s_{-2})$ and who are admitted to the nursing home 6-12 months after the survey has taken place (in calendar time T_1_, meaning between March and September 2013). Group 2 contains respondents interviewed 0-6 months before the nursing home admission (${\mathrm{in} s}_{-1})$ which means they are admitted to the nursing home 0-6 months after the survey (in T_0_). The same approach is followed for the other groups. The exact dates used for the classification of the groups of respondents can be found in Tables A4.1 and A4.2

Figure A4.1: Data structure

Panel a) Calendar time: time away from the survey T_k_

_
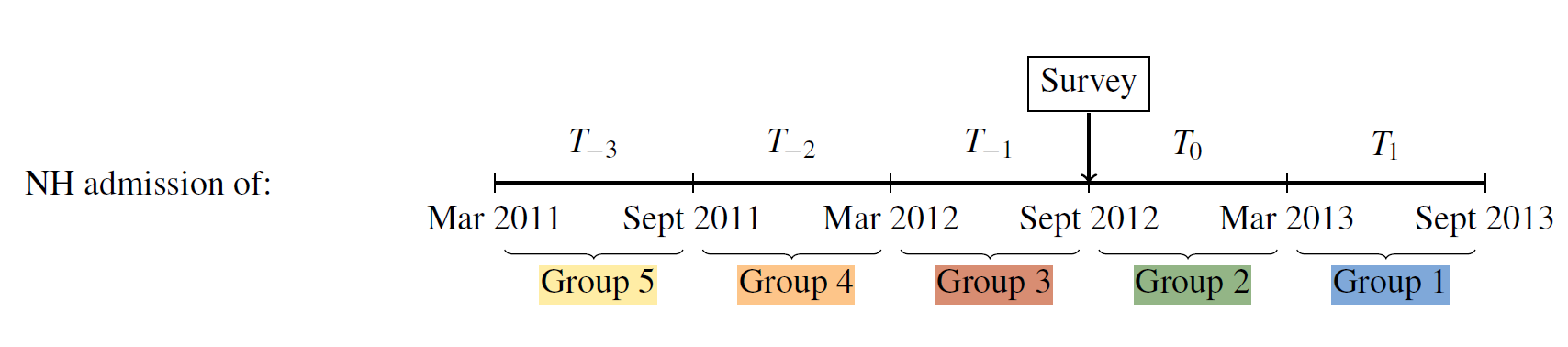
_

Panel b) Event time: time away from nursing home admission s_q_


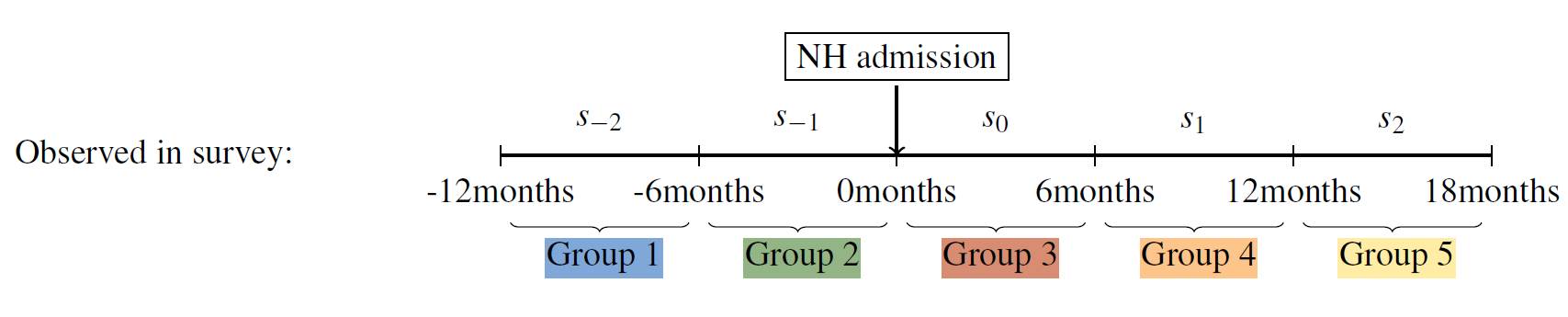


Note: Panel a) illustrates that observations are grouped by the timing of their nursing home (NH) admission relative to the timing of the survey. As the survey is conducted at one point in calendar time for everyone (September 2012), the grouping depends on the calendar time of the nursing home admission. For example, group 4 consists of all survey respondents who were admitted to a nursing home between September 2011 and February 2012. Panel b) illustrates at which event time the groups are observed in the survey, where event time is defined as time away from the nursing home admission. For example, group 4 is observed in the survey one period after the nursing home admission. One period corresponds to a six-month time span. The Figure depicts the situation for the respondents of the 2012 Health Monitor survey. The same structure applies for the 2016 respondents.

| **Group** | **Time since nursing home admission when completing survey** | **First day of 365+ days nursing home stay** |
| --- | --- | --- |
| 1 | -12 to -6 months (not yet admitted) | Between 01/03/2013 – 31/08/2013 |
| 2 | -6 to 0 months (not yet admitted) | Between 01/09/2012 – 28/02/2013 |
| 3 | 0-6 months (admitted) | Between 01/03/2012 – 31/08/2012 |
| 4 | 6-12 months (admitted) | Between 01/09/2011 – 29/02/2012 |
| 5 | 12-18 months (admitted) | Between 01/03/2011 – 31/08/2011 |

Table A4.1: Health monitor 2012, overview classification of groups of respondents

| **Group** | **Time since nursing home admission when completing survey** | **First day of 365+ days nursing home stay** |
| --- | --- | --- |
| 1 | -12 to -6 months (not yet admitted) | Between 01/03/2017 – 31/08/2017 |
| 2 | -6 to 0 months (not yet admitted) | Between 01/09/2016 – 28/02/2017 |
| 3 | 0-6 months (admitted) | Between 01/03/2016 – 31/08/2016 |
| 4 | 6-12 months (admitted) | Between 01/09/2015 – 29/02/2016 |
| 5 | 12-18 months (admitted) | Between 01/03/2015 – 31/08/2015 |

Table A4.2: Health monitor 2016, overview classification of groups of respondents

**Appendix 5 – Overview of variables included in the propensity score model**

| **Type** | **Included variables** |
| --- | --- |
| Health status and care needs | Type of nursing home care eligibility at T_-3_ but before nursing home admission  Using any home care in T_-4_  Hours of home care use in T_-4_  An indicator of prescription medication use in the calendar year before T_-3_ correlated with a nursing home admission: A02, A06, A10, A12, B01, B03, C01, C03, D02, G04, H02, J01, L01, L02, M01, M04, N05, N06, R01, R03, Y (Bakx et al., 2020a; Tenand et al., 2020)  Any hospital admission in T_-4_  Total length of stay at the hospital in T_-4_  Hospital admission by ISHTM category in T_-3_ and T_-4_  Indicators for age (everyone above 95 is grouped into a 95+ category) Gender  Indicator for short-term nursing home admission in T_-4_  Length of stay of short-term nursing home admission in T_-4_  Health insurance expenditure on hospital care in the calendar year before T_-3_  GP expenditure in the calendar year before T_-3_  Medication expenditure covered by the health insurance in the calendar year before T_-3_  Total health insurance expenditure in the calendar year before T_-3_. |
| Availability of nursing home care substitutes | Living with a partner in T_-4_  Becoming widowed in T_-4_ |
| Socioeconomic status | Household income quartiles in calendar year before T_-3_  Household wealth quartile in calendar year before T_-3_  Migration background |

**Appendix 6 – Propensity score models**

Table A6.1: Probability of a long-term nursing home admission at $T_{-3}$

|  | | **2012** | | **2016** | |
| --- | --- | --- | --- | --- | --- |
| Eligible for care package 1-4 $T_{-3}$ | | 2.825*** | (0.0368) | 5.317*** | (0.0854) |
| Eligible for care package 5&7 $T_{-3}$ | | 3.355*** | (0.0545) | 5.831*** | (0.0972) |
| Eligible for care package 6&8 $T_{-3}$ | | 3.573*** | (0.0722) | 5.978*** | (0.0927) |
| Eligible for care package 9 $T_{-3}$ | | 3.251*** | (0.0476) | 5.987*** | (0.204) |
| Eligible for care package 10 $T_{-3}$ | | 4.894*** | (0.415) | 5.776*** | (0.454) |
| Eligibility information missing $T_{-3}$ | | 0.546** | (0.248) | n.a. |  |
| Eligibility based on psychogeriatric condition $T_{-3}$ | | 0.461*** | (0.0370) | 0.275*** | (0.0553) |
| Home care $T_{-4}$ | | 0.0611* | (0.0325) | n.a. |  |
| Hours home care $T_{-4}$ | | 0.000360*** | (0.000) | n.a. |  |
| Expenditure home care in year before $T_{-3}$ | | n.a. |  | 0.00285 | (0.00203) |
| *Prescription medication (ATC3 code)* | |  |  |  |  |
|  | A02 at $T_{-4}$ | -0.00668 | -0.0286 | 0.0326 | (0.0391) |
|  | A06 at $T_{-4}$ | 0.0826*** | (0.0294) | -0.0285 | (0.0400) |
|  | A10 at $T_{-4}$ | -0.0236 | (0.0318) | 0.00880 | (0.0441) |
|  | A12 at $T_{-4}$ | -0.0140 | (0.0333) | -0.0741* | (0.0403) |
|  | B01 at $T_{-4}$ | 0.0359 | (0.0273) | -0.0745* | (0.0394) |
|  | B03 at $T_{-4}$ | 0.0140 | (0.0355) | -0.0125 | (0.0481) |
|  | C01 at $T_{-4}$ | -0.0395 | (0.0333) | -0.0641 | (0.0488) |
|  | C03 at $T_{-4}$ | -0.00791 | (0.0263) | 0.0570 | (0.0369) |
|  | D02 at $T_{-4}$ | 0.0335 | (0.0340) | -0.0483 | (0.0444) |
|  | G04 at $T_{-4}$ | 0.00245 | (0.0425) | 0.0977* | (0.0567) |
|  | H02 at $T_{-4}$ | 0.0422 | (0.0393) | -0.0567 | (0.0542) |
|  | J01 at $T_{-4}$ | -0.0328 | (0.0270) | -0.00281 | (0.0371) |
|  | L01 at $T_{-4}$ | 0.0353 | (0.121) | 0.311* | (0.161) |
|  | L02 at $T_{-4}$ | 0.228*** | (0.0884) | 0.0317 | (0.117) |
|  | M01 at $T_{-4}$ | 0.0404 | (0.0341) | -0.0118 | (0.0515) |
|  | M04 at $T_{-4}$ | 0.0524 | (0.0711) | 0.155 | (0.1000) |
|  | N05 at $T_{-4}$ | 0.128*** | (0.0346) | 0.0522 | (0.0440) |
|  | N06 at $T_{-4}$ | -0.104*** | (0.0307) | -0.238*** | (0.0392) |
|  | R01 at $T_{-4}$ | -0.115** | (0.0573) | 0.00777 | (0.0821) |
|  | R03 at $T_{-4}$ | -0.0595 | (0.0364) | -0.0437 | (0.0510) |
|  | Y at $T_{-4}$ | 0.131** | (0.0642) | -0.0306 | (0.0665) |
| Hospitalisation at $T_{-3}$ | | 0.0805 | (0.137) | 0.262 | (0.295) |
| Hospitalisation at $T_{-4}$ | | 0.650** | (0.264) | 0.0663 | (0.132) |
| Total LOS hospital at $T_{-3}$ | | 0.0191*** | (0.00197) | 0.0150*** | (0.00327) |
| Total LOS hospital at $T_{-4}$ | | 0.00643*** | (0.00202) | 0.0368*** | (0.00354) |
| Male | | 0.160*** | (0.0314) | 0.207*** | (0.0429) |
| Age 80-85 at $T_{-3}$ | | 0.0289 | (0.0368) | 0.0211 | (0.0520) |
| Age 85-90 at $T_{-3}$ | | 0.139*** | (0.0373) | 0.0253 | (0.0524) |
| Age 90-95 at $T_{-3}$ | | 0.189*** | (0.0445) | 0.0896 | (0.0601) |
| Age 95+ at $T_{-3}$ | | 0.411*** | (0.0782) | 0.348*** | (0.0998) |
| Dutch | | 0.0230 | (0.0427) | 0.0607 | (0.0540) |
| Alone living at $T_{-4}$ | | -0.444*** | (0.0531) | 0.228*** | (0.0629) |
| Living with partner at $T_{-4}$ | | -0.607*** | (0.0507) | 0.141** | (0.0598) |
| Partner in $T_{-4}$, alone in at $T_{-3}$ | | 0.500*** | (0.0953) | 0.489*** | (0.142) |
| Q2 income in year before $T_{-3}$ | | -0.311*** | (0.0365) | -0.378*** | (0.0530) |
| Q3 income in year before $T_{-3}$ | | -0.274*** | (0.0404) | -0.446*** | (0.0562) |
| Q4 income in year before $T_{-3}$ | | -0.340*** | (0.0436) | -0.453*** | (0.0606) |
| Q5 income in year before $T_{-3}$ | | -0.450*** | (0.0490) | -0.481*** | (0.0668) |
| Q2 wealth in year before $T_{-3}$ | | 0.0397 | (0.0377) | 0.0527 | (0.0526) |
| Q3 wealth in year before $T_{-3}$ | | 0.0948** | (0.0384) | 0.0738 | (0.0541) |
| Q4 wealth in year before $T_{-3}$ | | 0.0117 | (0.0415) | 0.118** | (0.0573) |
| Q5 wealth in year before $T_{-3}$ | | -0.0161 | (0.0454) | 0.0896 | (0.0611) |
| *Hospital admission diagnosis* | |  |  |  |  |
|  | Infection/parasites at $T_{-3}$ | 0.546** | (0.232) | -0.0471 | (0.367) |
|  | Neoplasms at $T_{-3}$ | 0.181 | (0.185) | 0.215 | (0.381) |
|  | Blood/bloodforming organs at $T_{-3}$ | -0.197 | (0.217) | 0.243 | (0.356) |
|  | Endocrine at $T_{-3}$ | 0.286 | (0.200) | 0.141 | (0.350) |
|  | Mental disorders at $T_{-3}$ | -0.0628 | (0.172) | 0.744** | (0.369) |
|  | Nervous system at $T_{-3}$ | 0.00253 | (0.185) | 0.272 | (0.340) |
|  | Eyes/adnexa at $T_{-3}$ | -0.406** | (0.168) | -0.263 | (0.359) |
|  | Ears at $T_{-3}$ | 0.519 | (0.578) | 1.277 | (1.389) |
|  | Circulatory at $T_{-3}$ | 0.319** | (0.144) | 0.537* | (0.305) |
|  | Respiratory at $T_{-3}$ | 0.426** | (0.169) | 0.418 | (0.320) |
|  | Digestive at $T_{-3}$ | -0.00221 | (0.166) | 0.201 | (0.326) |
|  | Skin at $T_{-3}$ | -0.182 | (0.288) | 0.592 | (0.459) |
|  | Musculoskeletal at $T_{-3}$ | -0.230 | (0.163) | 0.522 | (0.332) |
|  | Genitourinary at $T_{-3}$ | 0.312* | (0.176) | 0.532 | (0.326) |
|  | Congenital malformations at $T_{-3}$ | 0.518 | (1.136) | n.a. |  |
|  | Other at $T_{-3}$ | 0.0865 | (0.153) | 0.204 | (0.314) |
|  | Injury at $T_{-3}$ | 0.380*** | (0.146) | 0.783** | (0.304) |
|  | Factors influencing health services at $T_{-3}$ | -0.0861 | (0.168) | 0.125 | (0.371) |
|  | Infection/parasites at $T_{-4}$ | -0.582 | (0.354) | 0.259 | (0.267) |
|  | Neoplasms at $T_{-4}$ | -0.890*** | (0.296) | 0.495* | (0.290) |
|  | Blood/bloodforming organs at $T_{-4}$ | -0.789** | (0.332) | 0.127 | (0.253) |
|  | Endocrine at $T_{-4}$ | -0.346 | (0.315) | 0.714** | (0.281) |
|  | Mental disorders at $T_{-4}$ | -0.530* | (0.285) | 0.268 | (0.267) |
|  | Nervous system at $T_{-4}$ | -0.820*** | (0.304) | 0.275 | (0.218) |
|  | Eyes/adnexa at $T_{-4}$ | -0.538* | (0.277) | 0.292 | (0.252) |
|  | Ears at $T_{-4}$ | -0.556 | (0.598) | -0.125 | (0.693) |
|  | Circulatory at $T_{-4}$ | -0.502* | (0.271) | 0.629*** | (0.155) |
|  | Respiratory at $T_{-4}$ | -0.393 | (0.290) | 0.394** | (0.182) |
|  | Digestive at $T_{-4}$ | -0.724** | (0.284) | -0.0890 | (0.191) |
|  | Skin at $T_{-4}$ | -0.488 | (0.379) | -0.729* | (0.437) |
|  | Musculoskeletal at $T_{-4}$ | -0.854*** | (0.283) | 0.0584 | (0.209) |
|  | Genitourinary at $T_{-4}$ | -0.588** | (0.297) | 0.0946 | (0.199) |
|  | Other at $T_{-4}$ | -0.633** | (0.275) | 0.503*** | (0.177) |
|  | Injury at $T_{-4}$ | -1.021*** | (0.274) | 0.700*** | (0.156) |
|  | Factors influencing health services at $T_{-4}$ | -0.823*** | (0.279) | -0.0998 | (0.240) |
| *Other health related variables* | |  |  |  |  |
| Short NH stay at $T_{-4}$ | | -0.248*** | (0.0648) | -0.617*** | (0.125) |
| LOS short NH stay at $T_{-4}$ | | 0.0119*** | (0.000860) | -0.00348* | (0.00191) |
| HI expenditure GP in year before $T_{-3}$ | | 0.0573 | (0.0648) | 0.258*** | (0.0855) |
| HI expenditure hospital in year before $T_{-3}$ | | -0.0205*** | (0.00785) | -0.00644 | (0.0111) |
| HI expenditure total in year before $T_{-3}$ | | 0.0116* | (0.00702) | -0.000834 | (0.0101) |
| HI expenditure drugs in year before $T_{-3}$ | | -0.0127 | (0.0122) | -0.0327* | (0.0177) |
| Constant | | -3.145*** | (0.0783) | -5.807*** | (0.122) |
| N | | 65,741 |  | 54,597 |  |

Note: Calendar time $T_{-4}$ refers to the six-month period before nursing home (NH) admission of group 5 (the first admitted group), and calendar time $T_{-3}$ represents the six-month period of the first admitted group. We distinguish between six types of nursing home care eligibility grouping similar care packages. The definition of care packages can be found in Footnote 4. LOS stands for length of stay, and HI for health insurance. Q2 is short for second quintile and similarly for higher quintiles. Some information is not available (n.a.) for the 2016 cohort due to data recording changes for home care and care packages; or because no one was hospitalized with congenital malformations.

Figure A6.1: Propensity score distributions

| 1. 2012 sample | 1. 2016 sample |
| --- | --- |
| 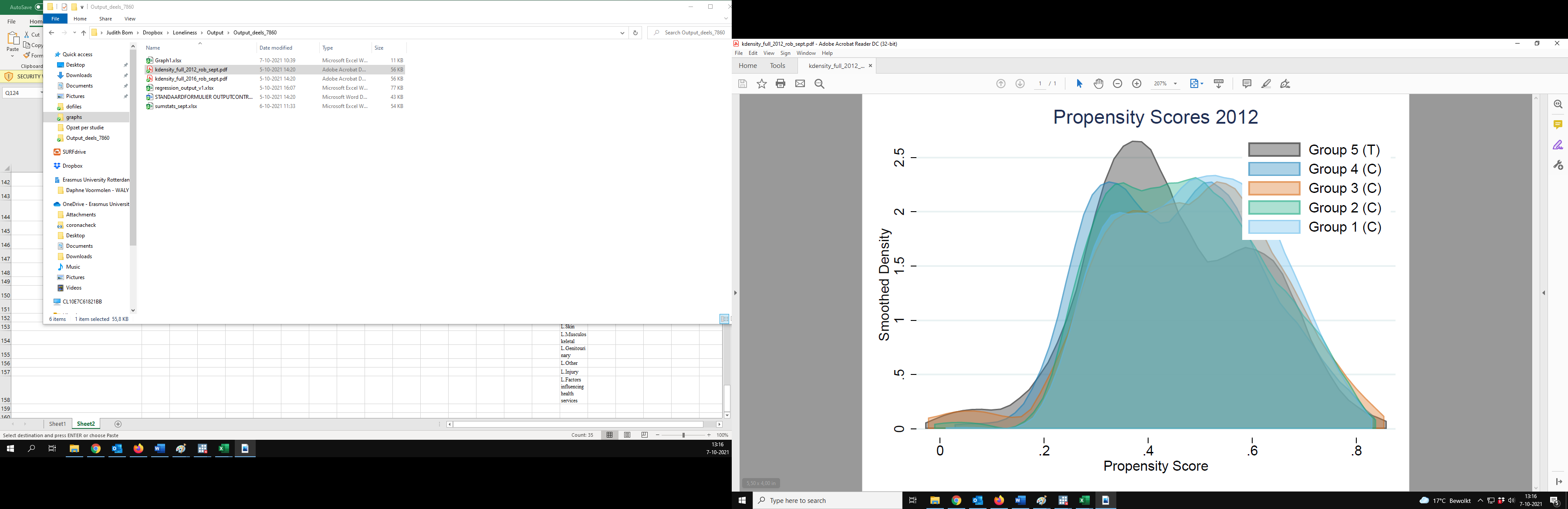 | 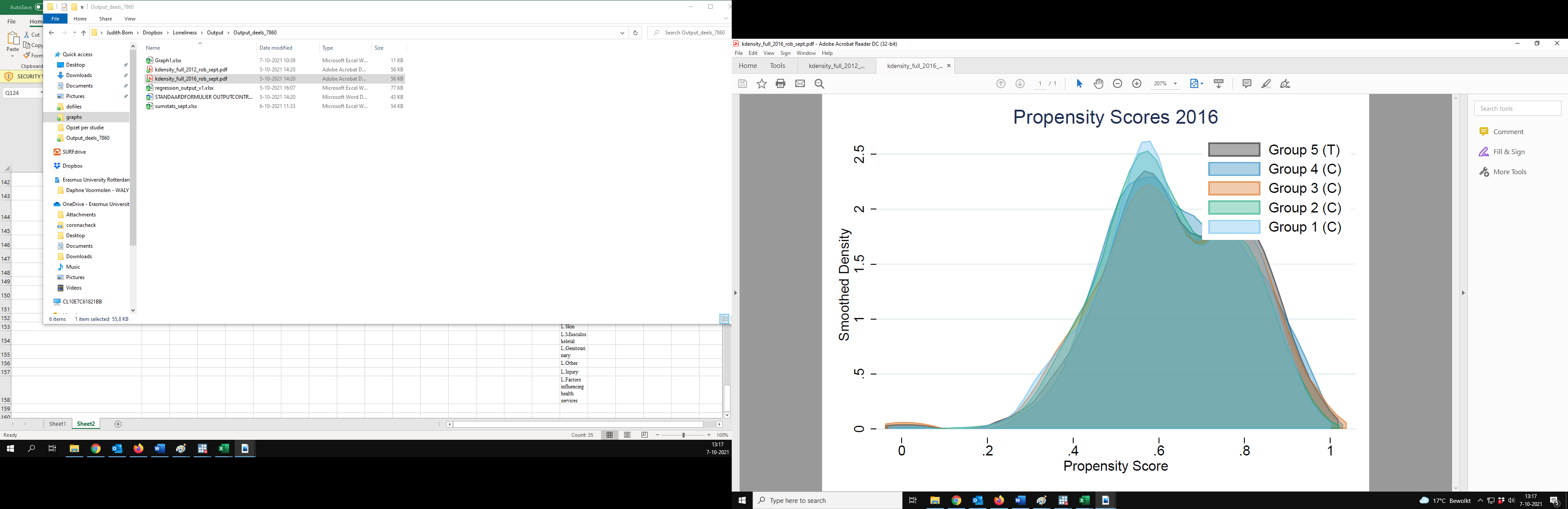 |

Note: Smoothed densities of propensity scores by groups. Group 5 represents the treatment group (T) in our setting, and groups 1-4 the control group (C).

**Appendix 7 – Comparability groups in health monitor sample**

To check comparability of the groups in our sample, Table A7.1 presents the descriptive statistics for the survey population before and after weighting the data. The tables show that the survey respondents in the different groups are already before matching comparable to each other. Table A7.2 presents a balancing table of the weighted health monitor sample by propensity score tercile. The table shows that for most characteristics, individuals are not meaningfully different from peers in the same propensity score tercile in other groups.

Table A7.1: Sample statistics of weighted and unweighted pooled health monitor sample

|  | **Weighted sample** | | | | | **Unweighted sample** | | | | |
| --- | --- | --- | --- | --- | --- | --- | --- | --- | --- | --- |
|  | **Mean group 1** | **Mean group 2** | **Mean group 3** | **Mean group 4** | **Mean group 5** | **Mean group 1** | **Mean group 2** | **Mean group 3** | **Mean group 4** | **Mean group 5** |
| Eligible for care packages 1-4 at $s_{0}$ | 0.33 | 0.35 | 0.32 | 0.32 | 0.35 | 0.42 | 0.43 | 0.41 | 0.42 | 0.44 |
| Eligible for care packages 5&7 at $s_{0}$ | 0.39 | 0.39 | 0.29 | 0.35 | 0.29 | 0.34 | 0.34 | 0.24 | 0.29 | 0.23 |
| Eligible for care packages 6&8 at $s_{0}$ | 0.24 | 0.17 | 0.21 | 0.14 | 0.20 | 0.21 | 0.14 | 0.16 | 0.10 | 0.15 |
| Eligible for care package 9 at $s_{0}$ | 0.04* | 0.09 | 0.18 | 0.20 | 0.16 | 0.03* | 0.08 | 0.17 | 0.19 | 0.15 |
| Eligibility based on psychogeriatric condition at $s_{0}$ | 0.51 | 0.54* | 0.41 | 0.47 | 0.36 | 0.47* | 0.49* | 0.36 | 0.39 | 0.30 |
| Age at $s_{0}$ | 85.9 | 85.6 | 85.6 | 85.2 | 84.9 | 85.9 | 85.6 | 85.6 | 85.0 | 84.6 |
| Male | 0.33 | 0.32 | 0.30 | 0.27 | 0.33 | 0.31 | 0.30 | 0.29 | 0.27 | 0.32 |
| Living with partner at $s_{-1}$ | 0.32 | 0.35 | 0.38 | 0.34 | 0.29 | 0.31 | 0.33 | 0.37 | 0.35 | 0.26 |
| A02 at $s_{-1}$ | 0.52 | 0.51 | 0.56 | 0.52 | 0.57 | 0.50 | 0.51 | 0.57 | 0.51 | 0.57 |
| A06 at $s_{-1}$ | 0.28 | 0.27 | 0.36 | 0.35 | 0.35 | 0.28 | 0.27 | 0.36 | 0.33 | 0.36 |
| A10 at $s_{-1}$ | 0.20 | 0.19 | 0.25 | 0.26 | 0.26 | 0.19 | 0.18 | 0.22 | 0.25 | 0.25 |
| A12 at $s_{-1}$ | 0.20 | 0.22 | 0.19 | 0.20 | 0.20 | 0.20 | 0.23 | 0.21 | 0.19 | 0.22 |
| B01 at $s_{-1}$ | 0.58 | 0.57 | 0.54 | 0.58 | 0.58 | 0.58 | 0.56 | 0.55 | 0.57 | 0.55 |
| B03 at $s_{-1}$ | 0.16 | 0.15 | 0.16 | 0.14 | 0.14 | 0.16 | 0.14 | 0.14 | 0.16 | 0.14 |
| C01 at $s_{-1}$ | 0.18 | 0.18 | 0.21 | 0.17 | 0.19 | 0.19 | 0.19 | 0.21 | 0.17 | 0.18 |
| C03 at $s_{-1}$ | 0.40 | 0.39 | 0.42 | 0.44 | 0.41 | 0.40 | 0.39 | 0.41 | 0.44 | 0.42 |
| D02 at $s_{-1}$ | 0.19 | 0.18 | 0.24 | 0.18 | 0.19 | 0.19 | 0.19 | 0.25 | 0.17 | 0.19 |
| G04 at $s_{-1}$ | 0.15 | 0.13 | 0.17 | 0.15 | 0.11 | 0.15 | 0.12 | 0.17 | 0.14 | 0.10 |
| H02 at $s_{-1}$ | 0.16 | 0.13 | 0.18 | 0.13 | 0.13 | 0.15 | 0.13 | 0.19 | 0.11 | 0.13 |
| J01 at $s_{-1}$ | 0.46 | 0.38 | 0.42 | 0.45 | 0.33 | 0.45 | 0.38 | 0.41 | 0.44 | 0.34 |
| M01/M04 at $s_{-1}$ | 0.20 | 0.19 | 0.22 | 0.18 | 0.23 | 0.20 | 0.19 | 0.21 | 0.19 | 0.23 |
| R03 at $s_{-1}$ | 0.18 | 0.15 | 0.18 | 0.19 | 0.11 | 0.18 | 0.16 | 0.19 | 0.18 | 0.12 |
| Other drugs at $s_{-1}$ | 0.73 | 0.62 | 0.88* | 0.82 | 0.56 | 0.73 | 0.63 | 0.85 | 0.80 | 0.59 |
| Hospitalisation | 0.39 | 0.43 | 0.51 | 0.38 | 0.34 | 0.34 | 0.37 | 0.43 | 0.33 | 0.28 |
| Hospitalisation at $s_{-1}$ | 0.33 | 0.27 | 0.24 | 0.29 | 0.29 | 0.30 | 0.24 | 0.19 | 0.24 | 0.23 |
| Total LOS hospital | 5.32 | 6.52 | 8.50 | 6.40 | 4.95 | 4.36 | 5.04 | 6.72 | 4.91 | 3.83 |
| Total LOS hospital at $s_{-1}$ | 4.01 | 2.88 | 4.17 | 5.13 | 3.29 | 3.42 | 2.35 | 3.07 | 3.70 | 2.33 |
| Dutch | 0.89 | 0.90 | 0.90 | 0.88 | 0.89 | 0.89 | 0.90 | 0.90 | 0.88 | 0.89 |
| Alone living at $s_{-1}$ | 0.62 | 0.57 | 0.50 | 0.58 | 0.67 | 0.63 | 0.58 | 0.53 | 0.58 | 0.69 |
| Living with partner at $s_{-1}$ | 0.32 | 0.35 | 0.38 | 0.34 | 0.29 | 0.31 | 0.33 | 0.37 | 0.35 | 0.26 |
| Q1 income in year before $s_{0}$ | 0.24 | 0.19* | 0.28 | 0.21* | 0.39 | 0.23 | 0.19* | 0.273 | 0.19* | 0.37 |
| Q2 income in year before $s_{0}$ | 0.20 | 0.19 | 0.18 | 0.20 | 0.15 | 0.21 | 0.20 | 0.18 | 0.20 | 0.16 |
| Q3 income in year before $s_{0}$ | 0.19 | 0.18 | 0.18 | 0.19 | 0.14 | 0.19 | 0.19 | 0.18 | 0.19 | 0.14 |
| Q4 income in year before $s_{0}$ | 0.18 | 0.20 | 0.20 | 0.19 | 0.14 | 0.18 | 0.20 | 0.20 | 0.20 | 0.14 |
| Q5 income in year before $s_{0}$ | 0.20 | 0.23 | 0.16 | 0.22 | 0.18 | 0.20 | 0.23 | 0.16 | 0.22 | 0.20 |
| Q1 wealth in year before $s_{0}$ | 0.18 | 0.17 | 0.13 | 0.18 | 0.18 | 0.18 | 0.17 | 0.13 | 0.19 | 0.19 |
| Q2 wealth in year before $s_{0}$ | 0.22 | 0.19 | 0.23 | 0.22 | 0.24 | 0.23 | 0.19 | 0.23 | 0.20 | 0.22 |
| Q3 wealth in year before $s_{0}$ | 0.21 | 0.19 | 0.26 | 0.23 | 0.21 | 0.21 | 0.18 | 0.27 | 0.23 | 0.22 |
| Q4 wealth in year before $s_{0}$ | 0.18 | 0.23 | 0.17 | 0.17 | 0.19 | 0.18 | 0.23 | 0.18 | 0.18 | 0.20 |
| Q5 wealth in year before $s_{0}$ | 0.20 | 0.23 | 0.21 | 0.20 | 0.18 | 0.20 | 0.23 | 0.19 | 0.21 | 0.18 |
| Short NH stay at $s_{-1}$ | 0.04 | 0.06 | 0.10 | 0.12 | *^1^ | 0.04 | 0.06 | 0.10 | 0.10 | 0.08 |
| LOS short NH stay at $s_{-1}$ | 1.65 | 4 | 4.51 | 10 | 3.60 | 1.52 | 3.31 | 3.83 | 7.93 | 5.11 |
| HI expenditure GP in year before $s_{0}$ | 0.36 | 0.34 | 0.43 | 0.32 | 0.33 | 0.36 | 0.33 | 0.40 | 0.30 | 0.33 |
| HI expenditure hospital in year before $s_{0}$ | 3482 | 3072 | 5067 | 3316 | 3564 | 3367 | 3123 | 4671 | 3135 | 3542 |
| HI expenditure total in year before $s_{0}$ | 6632 | 5985 | 8758 | 6408 | 6821 | 6451 | 6002 | 8261 | 6078 | 6854 |
| HI expenditure drugs in year before $s_{0}$ | 1087 | 1016 | 1328 | 1139 | 1081 | 1081 | 1022 | 1336 | 1098 | 1131 |
| N | 1,048 | 729 | 187 | 168 | 123 | 1,048 | 729 | 187 | 168 | 123 |

Note: Differences between groups are calculated using standardized differences between group 5 and one of the other groups, * standardized difference > 0.25 following the threshold of Stuart et al. (2013). Event time $s_{0}$ refers to the period of the nursing home (NH) admission, and $s_{-1}$to the six-month period before nursing home admission. We distinguish between four types of nursing home care eligibility grouping similar care packages. The definition of care packages can be found in Footnote 4. LOS stands for length of stay, and HI for health insurance. Q1 is short for first quintile and similarly for higher quintiles. ^1^ Result not reported for confidentiality reasons.

Table A7.2: Sample statistics of weighted health monitor sample by propensity score tercile

| **1st tercile** | **Mean group 1** | **Mean group 2** | **Mean group 3** | **Mean group 4** | **Mean group 5** |
| --- | --- | --- | --- | --- | --- |
| Eligible for care packages 1-4 at $s_{0}$ | 0.87 | 0.84 | 0.82 | 0.78 | 0.79 |
| Eligible for care packages 5&7 at $s_{0}$ | 0.04 | 0.06 | 0.03 | 0.06 | 0.00 |
| Eligible for care packages 6&8 at $s_{0}$ | 0.08 | 0.05 | 0.02 | 0.02 | 0.02 |
| Eligible for care package 9 at $s_{0}$ | 0.01* | 0.05* | 0.12 | 0.14 | 0.18 |
| Eligibility based on psychogeriatric condition at $s_{0}$ | 0.24* | 0.26* | 0.23 | 0.18 | 0.10 |
| Age at $s_{0}$ | 85.9* | 85.9* | 86.2* | 85.1* | 83.3 |
| Male | 0.21 | 0.25 | 0.37 | 0.24 | 0.31 |
| N | 309 | 245 | 70 | 67 | 58 |
| **2nd tercile** | **Mean group 1** | **Mean group 2** | **Mean group 3** | **Mean group 4** | **Mean group 5** |
| Eligible for care packages 1-4 at $s_{0}$ | 0.30 | 0.28 | 0.25 | 0.20 | 0.16 |
| Eligible for care packages 5&7 at $s_{0}$ | 0.48 | 0.52 | 0.37 | 0.42 | 0.50 |
| Eligible for care packages 6&8 at $s_{0}$ | 0.21 | 0.12 | 0.13 | 0.09 | 0.19 |
| Eligible for care package 9 at $s_{0}$ | 0.01* | 0.08 | 0.25 | 0.28 | 0.15 |
| Eligibility based on psychogeriatric condition at $s_{0}$ | 0.60 | 0.65 | 0.49 | 0.52 | 0.59 |
| Age at $s_{0}$ | 85.3 | 85.1 | 84.7 | 84.8 | 85.0 |
| Male | 0.32 | 0.28 | 0.23 | 0.33 | 0.29 |
| N | 351 | 250 | 61 | 55 | 31 |
| **3rd tercile** | **Mean group 1** | **Mean group 2** | **Mean group 3** | **Mean group 4** | **Mean group 5** |
| Eligible for care packages 1-4 at $s_{0}$ | 0.14 | 0.16 | 0.09 | 0.12 | 0.11 |
| Eligible for care packages 5&7 at $s_{0}$ | 0.47 | 0.47 | 0.38 | 0.49 | 0.39 |
| Eligible for care packages 6&8 at $s_{0}$ | 0.32 | 0.27 | 0.38 | 0.25 | 0.36 |
| Eligible for care package 9 at $s_{0}$ | 0.07 | 0.11 | 0.15 | 0.15 | 0.14 |
| Eligibility based on psychogeriatric condition at $s_{0}$ | 0.57 | 0.60* | 0.45 | 0.63* | 0.42 |
| Age at $s_{0}$ | 86.4 | 85.9 | 85.9 | 85.7 | 86.1 |
| Male | 0.38 | 0.38 | 0.32 | 0.25 | 0.38 |
| N | 382 | 231 | 56 | 46 | 33 |

Note: Individuals are grouped in terciles dependent on their propensity score. Differences between groups are calculated using standardized differences between group 5 and one of the other groups, * standardized difference > 0.25 following the threshold of Stuart et al. (2013). Event time $s_{0}$ refers to the period of the nursing home (NH) admission. We distinguish between four types of nursing home care eligibility grouping similar care packages. The definition of care packages can be found in Footnote 4. This table presents means for a selection of variables, a table including all variables used for matching is available upon request.

**Appendix 8 - Comparability in pre-admission trends across groups**

| **Health Care spending in basic insurance package** | |
| --- | --- |
| Mean annual health care spending of individuals within 6 months intervals before nursing home admission (2012). Group 1, 3 and 5 | Mean annual health care spending of individuals within 6 months intervals before nursing home admission (2012). Group 2 and 4 |
|  |  |
| Mean annual health care spending of individuals within 6 months intervals before nursing home admission (2016). Group 1, 3 and 5 | Mean annual health care spending of individuals within 6 months intervals before nursing home admission (2016). Group 2 and 4 |
|  |  |

Note: As we are using annual data trends are not similar for all groups. Time 0 represents the average health care spending in the year of admission, for example for group 1 who is admitted between March and September 2013, the 2013 expenditures. At time -1 we for this group use September 2012-March 2013 as reference and use the 2012 data, same goes for time -2 were we use the March 2012-September 2012 data. For group 2 (admitted between September 2012-March 2013) we use the 2012 data for both Time 0 and Time -1. Hence, we can only compare trends between group 1,3 and 5 and group 2 and 4.

| **Home care** | |
| --- | --- |
| Proportion of individuals within cohort are making use of home care within 6 months intervals before nursing home admission (2012) | Average hours of home care per cohort in 6 months intervals before nursing home admission (2012) |
|  |  |

**Appendix 9 – Well-being and functional limitation scores by group**

Table A9.1: Well-being and physical health scores by group for the pooled sample (weighted)

| **Health Monitor 2012+2016, weighted** | **Group 1** | **Group 2** | **Group 3** | **Group 4** | **Group 5** |
| --- | --- | --- | --- | --- | --- |
|  | **Mean** | **Mean** | **Mean** | **Mean** | **Mean** |
| Loneliness | 5.19 | 5.30 | 6.33 | 5.51 | 5.61 |
| Social loneliness | 2.26 | 2.30 | 2.65 | 2.22 | 2.38 |
| Emotional loneliness | 2.96 | 3.04 | 3.69 | 3.37 | 3.28 |
| Depression & anxiety | 22.66 | 25.01 | 26.39 | 25.56 | 24.97 |
| Inadequate control over life^1^ | 22.27 | 24.96 | 27.33 | 26.36 | 26.73 |
| Functional limitations | 2.58 | 3.10 | 3.55 | 3.28 | 3.88 |
| Observations | 997 | 685 | 163 | 148 | 106 |

Note: Observations differ by outcome as sample is not restricted to availability of all outcomes. ^1^ The outcome inadequate control over life is only reported for the 2016 sample.

**Appendix 10 – Regression results**

Table A10.1: Regression results pooled sample

|  | **Lonely** | **Social Loneliness** | **Emotional loneliness** | **Anxiety and depression** | **Inadequate control over life^1^** | **Functional limitations** |
| --- | --- | --- | --- | --- | --- | --- |
| *Time since nursing home admission* |  |  |  |  |  |  |
| -12 to -6 months (not yet admitted) | -0.276 | -0.178* | -0.0916 | -1.874*** | -2.202*** | -0.361*** |
|  | (0.203) | (0.108) | (0.131) | (0.505) | (0.666) | (0.105) |
| 0-6 months (admitted) | 1.107*** | 0.377** | 0.726*** | 1.507* | 2.274*** | 0.461*** |
|  | (0.362) | (0.191) | (0.227) | (0.852) | (0.871) | (0.154) |
| 6-12 months (admitted) | 0.125 | -0.0904 | 0.285 | 0.590 | 1.150 | 0.179 |
|  | (0.377) | (0.204) | (0.241) | (1.005) | (1.282) | (0.190) |
| 12-18 months (admitted) | 0.0586 | 0.0586 | 0.0247 | -0.0255 | 1.648 | 0.728*** |
|  | (0.440) | (0.221) | (0.283) | (0.951) | (1.177) | (0.181) |
| Married at T_0_ | -1.558*** | -0.406*** | -1.167*** | 0.450 | 0.243 | -0.0485 |
|  | (0.201) | (0.107) | (0.130) | (0.489) | (0.473) | (0.100) |
| Antithrombotics at $s_{-1}$ | -0.512** | -0.270** | -0.232* | -0.170 | -0.695 | 0.0108 |
|  | (0.205) | (0.111) | (0.132) | (0.512) | (0.676) | (0.101) |
| Drug for acid-related disorders at $s_{-1}$ | 0.294 | -0.0272 | 0.357*** | 1.923*** | 1.819*** | 0.369*** |
|  | (0.209) | (0.111) | (0.135) | (0.526) | (0.672) | (0.102) |
| Drugs for diabetes$\mathrm{at}s_{-1}$ | -0.0228 | -0.0952 | 0.0195 | -0.111 | -0.400 | 0.176 |
|  | (0.254) | (0.131) | (0.160) | (0.610) | (0.767) | (0.114) |
| Drugs for obstructive airway diseases at $s_{-1}$ | -0.0553 | 0.0248 | -0.0905 | 0.000291 | -0.416 | 0.168 |
|  | (0.282) | (0.146) | (0.181) | (0.643) | (0.974) | (0.127) |
| Eligible for care package 1-4 at $s_{0}$ | 1.093 | 0.0408 | 1.124 | -0.742 | -5.029*** | -0.786 |
|  | (1.477) | (0.650) | (0.934) | (4.233) | (0.661) | (0.544) |
| Eligible for care package 5&7$\mathrm{at}s_{0}$ | 1.423 | 0.188 | 1.319 | 1.898 | -3.380*** | -0.155 |
|  | (1.479) | (0.652) | (0.936) | (4.242) | (0.685) | (0.547) |
| Eligible for care package 6&8 at $s_{0}$ | 1.248 | 0.133 | 1.138 | 0.902 | -4.518*** | -0.251 |
|  | (1.486) | (0.657) | (0.939) | (4.254) | (0.716) | (0.547) |
| Eligible for rehabilitative care at $s_{0}$ | 0.963 | -0.0144 | 1.050 | 0.834 | -5.216*** | -0.431 |
|  | (1.503) | (0.670) | (0.953) | (4.315) | (1.577) | (0.562) |
| Hospitalization$\mathrm{at}s_{0}$ | -0.315* | -0.0594 | -0.326*** | -0.705 | -0.967** | -0.111 |
|  | (0.182) | (0.0989) | (0.118) | (0.446) | (0.457) | (0.0911) |
| Male | 0.137 | 0.183* | -0.0462 | -1.743*** | -0.434 | -0.250** |
|  | (0.194) | (0.105) | (0.127) | (0.470) | (0.465) | (0.100) |
| Aged 80-85 $\mathrm{at}s_{0}$ | 0.415 | 0.185 | 0.227 | -0.836 | -0.405 | -0.0142 |
|  | (0.279) | (0.151) | (0.181) | (0.703) | (0.711) | (0.140) |
| Aged 85-90 $\mathrm{at}s_{0}$ | -0.0295 | 0.0226 | -0.0911 | -1.335* | 0.146 | 0.204 |
|  | (0.269) | (0.146) | (0.173) | (0.685) | (0.650) | (0.137) |
| Aged 90-95 $\mathrm{at}s_{0}$ | 0.0482 | -0.137 | 0.174 | -1.518** | 0.621 | 0.447*** |
|  | (0.291) | (0.156) | (0.187) | (0.749) | (0.701) | (0.149) |
| Aged 95+ $\mathrm{at}s_{0}$ | -0.230 | -0.156 | -0.0231 | -1.405 | 0.662 | 1.493*** |
|  | (0.519) | (0.263) | (0.354) | (1.203) | (1.242) | (0.249) |
| Constant | 4.794*** | 2.482*** | 2.304** | 25.19*** | 29.01*** | 3.171*** |
|  | (1.503) | (0.667) | (0.952) | (4.296) | (1.090) | (0.561) |
| N | 1,736 | 1,721 | 1,647 | 1,895 | 768 | 2,037 |

Note: * p < 0.10, ** p < 0.05, *** p<0.01; Results are weighted. We distinguish between four types of nursing home care eligibility grouping similar care packages. The definition of care packages can be found in Footnote 4. ^1^ The outcome inadequate control over life is only reported for the 2016 sample.

**Appendix 11 – Robustness checks**

Figure A11.1: Robustness checks – Results per survey

Note: Estimates of $\hat{\delta_{q}}$ and their 90, 95, and 99 confidence intervals. The following results are presented: “2012” main results for the 2012 sample, “2016” main results for the 2016 sample.

Table A11.1: Logit models using binarized outcomes (weighted), margins

|  | **Lonely** | **Social Loneliness** | **Emotional loneliness** | **Anxiety and depression** | **Inadequate control over life^1^** |
| --- | --- | --- | --- | --- | --- |
|  | **Margins** | **Margins** | **Margins** | **Margins** | **Margins** |
| *Time since nursing home admission* |  |  |  |  |  |
| -12 to -6 months (not yet admitted) | -0.0348 | -0.0617** | -0.0227 | -0.0992*** | -0.153** |
| -6 to 0 months (not yet admitted) | Ref. | Ref. | Ref. | Ref. | Ref. |
| 0-6 months (admitted) | 0.0530 | 0.112** | 0.146*** | 0.0754 | 0.125 |
| 6-12 months (admitted) | 0.0103 | -0.0810 | 0.0359 | 0.0247 | 0.0686 |
| 12-18 months (admitted) | -0.00608 | 0.00878 | 0.0206 | 0.0260 | 0.137 |
| N | 1,736 | 1,721 | 1,647 | 1,895 | 768 |

Note: All well-being scales are transformed into binary variables were a 1 represents a worse health score. This refers to reporting average to high general, emotional or social loneliness score; having a medium to high risk of depression/anxiety and inadequate control over life. Including controls: male, age, married, atc B01; atc R03; atc A02, atc_A10, ZZP-score and hospitalization. * p < 0.10, ** p < 0.05, *** p<0.01. Results are weighted. ^1^ The outcome inadequate control over life is only reported for the 2016 sample.

Table A11.2: Regression results 2012 sample

|  | **Lonely** | **Social Loneliness** | **Emotional loneliness** | **Anxiety and depression** |
| --- | --- | --- | --- | --- |
| *Time since nursing home admission* |  |  |  |  |
| -12 to -6 months (not yet admitted) | -0.229 | -0.108 | -0.0887 | -1.895*** |
|  | (0.268) | (0.142) | (0.172) | (0.685) |
| -6 to 0 months (not yet admitted) | Ref. | Ref. | Ref. | Ref. |
|  |  |  |  |  |
| 0-6 months (admitted) | 1.355*** | 0.473** | 0.884*** | 0.909 |
|  | (0.427) | (0.229) | (0.273) | (1.010) |
| 6-12 months (admitted) | -0.110 | -0.317 | 0.293 | -0.267 |
|  | (0.451) | (0.230) | (0.293) | (1.121) |
| 12-18 months (admitted) | -0.360 | -0.146 | -0.187 | -0.983 |
|  | (0.509) | (0.259) | (0.338) | (1.146) |
| N | 979 | 969 | 931 | 1,058 |

Note: Including controls: male, age, married, atc B01; atc R03; atc A02, atc_A10, care package scores and hospitalization. * p < 0.10, ** p < 0.05, *** p<0.01. Results are weighted.

Table A11.3: Regression results 2016 sample

|  | **Lonely** | **Social Loneliness** | **Emotional loneliness** | **Anxiety and depression** | **Inadequate control over life** |
| --- | --- | --- | --- | --- | --- |
| *Time since nursing home admission* |  |  |  |  |  |
| -12 to -6 months (not yet admitted) | -0.595 | -0.344* | -0.271 | -2.252** | -2.202*** |
|  | (0.380) | (0.207) | (0.249) | (1.007) | (0.666) |
| -6 to 0 months (not yet admitted) | Ref. | Ref. | Ref. | Ref. | Ref. |
|  |  |  |  |  |  |
| 0-6 months (admitted) | 0.839 | 0.370 | 0.448 | 3.042* | 2.274*** |
|  | (0.709) | (0.352) | (0.424) | (1.577) | (0.871) |
| 6-12 months (admitted) | 0.740 | 0.458 | 0.376 | 2.183 | 1.150 |
|  | (0.685) | (0.376) | (0.431) | (1.918) | (1.282) |
| 12-18 months (admitted) | 0.932 | 0.445 | 0.447 | 2.046 | 1.648 |
|  | (0.777) | (0.383) | (0.492) | (1.662) | (1.177) |
| N | 757 | 752 | 716 | 837 | 768 |

Note: Including controls: male, age, married, atc B01; atc R03; atc A02, atc_A10, care package score and hospitalization. * p < 0.10, ** p < 0.05, *** p<0.01. Results are weighted.

Table A11.4: Regression results November sample

|  | **Lonely** | **Social Loneliness** | **Emotional loneliness** | **Anxiety and depression** | **Inadequate control over life^1^** |
| --- | --- | --- | --- | --- | --- |
| *Time since nursing home admission* |  |  |  |  |  |
| -12 to -6 months (not yet admitted) | -0.285 | -0.0923 | -0.197 | -1.541*** | -1.579*** |
|  | (0.186) | (0.0999) | (0.121) | (0.455) | (0.571) |
| -6 to 0 months (not yet admitted) | Ref. | Ref. | Ref. | Ref. | Ref. |
|  |  |  |  |  |  |
| 0-6 months (admitted) | 0.710** | 0.279* | 0.387** | 0.952 | 2.327*** |
|  | (0.295) | (0.152) | (0.187) | (0.721) | (0.657) |
| 6-12 months (admitted) | 0.492 | 0.0245 | 0.581** | 0.920 | 1.046 |
|  | (0.376) | (0.205) | (0.228) | (0.901) | (1.103) |
| 12-18 months (admitted) | -0.0668 | -0.0453 | -0.0203 | 0.734 | 2.433** |
|  | (0.387) | (0.197) | (0.250) | (0.953) | (1.075) |
| N | 1,977 | 1,962 | 1,869 | 2,150 | 894 |

Note: Including controls: male, age, married, atc B01; atc R03; atc A02, atc_A10, care package score and hospitalization. * p < 0.10, ** p < 0.05, *** p<0.01. Results are weighted. ^1^. The outcome inadequate control over life is only reported for the 2016 sample.

Table A11.5: Regression results excluding individuals who enter for rehabilitative care (zzp9)

|  | **Lonely** | **Social Loneliness** | **Emotional loneliness** | **Anxiety and depression** | **Inadequate control over life^1^** |
| --- | --- | --- | --- | --- | --- |
| *Time since nursing home admission* |  |  |  |  |  |
| -12 to -6 months (not yet admitted) | -0.365* | -0.202* | -0.149 | -1.783*** | -2.163*** |
|  | (0.209) | (0.111) | (0.135) | (0.511) | (0.690) |
| -6 to 0 months (not yet admitted) | Ref. | Ref. | Ref. | Ref. | Ref. |
|  |  |  |  |  |  |
| 0-6 months (admitted) | 0.561 | 0.206 | 0.360 | 1.651* | 2.312** |
|  | (0.424) | (0.213) | (0.258) | (0.935) | (0.900) |
| 6-12 months (admitted) | 0.191 | -0.0640 | 0.300 | 1.406 | 1.318 |
|  | (0.438) | (0.228) | (0.272) | (1.096) | (1.265) |
| 12-18 months (admitted) | -0.0537 | 0.0379 | -0.0368 | 0.144 | 1.840 |
|  | (0.490) | (0.240) | (0.308) | (1.025) | (1.192) |
| N | 1,600 | 1,588 | 1,520 | 1,754 | 750 |

Note: Including controls: male, age, married, atc B01; atc R03; atc A02, atc_A10, care package score and hospitalization. * p < 0.10, ** p < 0.05, *** p<0.01. Results are weighted. ^1^ The outcome inadequate control over life is only reported for the 2016 sample.

Table A11.6: Regression results excluding individuals who stay for at least 180 days

|  | **Lonely** | **Social Loneliness** | **Emotional loneliness** | **Anxiety and depression** | **Inadequate control over life^1^** |
| --- | --- | --- | --- | --- | --- |
| *Time since nursing home admission* |  |  |  |  |  |
| -12 to -6 months (not yet admitted) | -0.252 | -0.160 | -0.0865 | -1.874*** | -2.036*** |
|  | (0.205) | (0.108) | (0.132) | (0.509) | (0.679) |
| -6 to 0 months (not yet admitted) | Ref. | Ref. | Ref. | Ref. | Ref. |
|  |  |  |  |  |  |
| 0-6 months (admitted) | 1.139*** | 0.389** | 0.736*** | 1.458* | 2.281** |
|  | (0.362) | (0.190) | (0.227) | (0.838) | (0.897) |
| 6-12 months (admitted) | 0.0651 | -0.127 | 0.246 | 0.854 | 1.072 |
|  | (0.385) | (0.205) | (0.248) | (1.002) | (1.269) |
| N | 1,662 | 1,648 | 1,576 | 1,818 | 751 |

Note: Including controls: male, age, married, atc B01; atc R03; atc A02, atc_A10, care package score and hospitalization. * p < 0.10, ** p < 0.05, *** p<0.01. Results are weighted. ^1^ The outcome inadequate control over life is only reported for the 2016 sample.

**Appendix 12 – Extended explanation of the robustness checks regarding potential confounding**

The doubly robust procedure, used for our main analyses, mitigates the differences between the five cohorts at the time of the admission through the inverse probability weighting and through adding controls to the event study regression. Furthermore, the results discussed in Section 5.2 indicate that all five cohorts have the same trend in health care use before the nursing home admission.

However, gradual changes, e.g. in health status, might play a role in the period around the nursing home admission. In subsection 6.2, we therefore explored the potential role of such gradual changes. For brevity we only discussed the results of these analyses in the main text. This Appendix provides more insight the reasons for performing these robustness checks. Specifically, two steps were taken: First, we extended the length of the pre-trend to four periods (i.e. 2 year) and used this longer pre-trend to study the potential role of confounding on the interpretation of our estimates by extrapolation of the pre-trend (subsection A12.1). Second, we explicitly explored how physical health, which is a potential confounder and not controlled for in the event study regression because it may be endogenous to well-being, evolves over time (subsection A12.2).

**A12.1 Modelling the impact of unobserved confounding using longer pre-trends in well-being**

Extending the number of pre-event periods from two to four 6-month periods (Main text: Figure 5, top panel) confirmed our conclusion from the main analyses, namely that loneliness rates are stable in the period before the admission. At the same time, there is an increase in anxiety and depression that halts around 6 months after someone is admitted to a nursing home, and there is a gradual loss of control throughout the period before the nursing home admission.

These pre-trends may be an indicator of unobserved confounding (Freyaldenhoven et al., 2021). This confounding may lead to a clear trend in the pre-event periods, for example a linear increase in the risk of anxiety and depression in our case. Even though the confounding variable causing this linear increasing pre-trend is unobserved, we may assume that the unobserved variable continues influencing anxiety and depression in the same way after the admission (Doyle et al., 2018; Freyaldenhoven et al., 2021). Hence, by extrapolating the trend in the pre-trend period, we can model the distortion of the event study estimates caused by the confounder and thus better understand the implications that this confounding may have for the interpretation of the event study estimates. This is done by imposing a linear trend on the observations that is based on the pre-event estimates only. This trend is used to rescale the estimates to show deviations from the trend line instead of differences compared to period $s_{-1}$ (Doyle et al., 2018; Freyaldenhoven et al., 2021).

In Figure 5, linear trends are used to rescale the estimates. These linear trends fit the trend in the pre-event periods entirely for all outcomes, except for the outcome loss of control in event time $s_{-2}$, where the coefficient is still borderline significant at 5%. For this reason, we also fit a quadratic trend as an alternative robustness check. The results using a quadratic trend for the outcome inadequate control over life are depicted in Figure A12.1 below. Additionally, Figure A12.2 presents graphs for emotional and social loneliness as Figure 5 in the main texts only depicts overall loneliness results.

Figure A12.1: Effect of a nursing home admission on inadequate control over life (2016 sample) while (lower panel) rescaling estimates to indicate deviations from a quadratic trend line

Note: Top panel: Estimates of $\hat{\delta_{q}}$ and their 90, 95, and 99 confidence intervals. Middle panel: Estimates of $\hat{\delta_{q}}$ and their 90, 95, and 99 confidence intervals with a fitted quadratic pre-trend. Bottom panel: rescaled estimates as deviations from the trend line estimated in the middle panel.

Figure A12.2: Effect of a nursing home admission on social and emotional loneliness (pooled sample) while (lower panel) rescaling estimates to indicate deviations from the trend line

Note: Top panel: Estimates of $\hat{\delta_{q}}$ and their 90, 95, and 99 confidence intervals. Middle panel: Estimates of $\hat{\delta_{q}}$ and their 90, 95, and 99 confidence intervals with a fitted linear pre-trend. Bottom panel: rescaled estimates as deviations from the trend line estimated in the middle panel.

**A12.2 Functional limitations**

Second, we investigated trends in one important potential confounder: functional limitations, which is a major reason for long-term care use (de Meijer et al., 2009, 2013) and is measured through a 7-item list^[[1]](#footnote-1)^ of tasks performed everyday life. An increasing trend in functional limitations might be driving the pre-trends in our well-being estimates. Checking whether the trend in functional limitations is linear and increasing aids in assessing whether the rescaling of the event-study coefficients by extrapolating a linear trend (as done in Figure 5) may capture the potential confounding role of functional limitations.

Furthermore, analyzing functional limitations allowed us to check whether the model picks up a trend that continues in the post-event period when we would be certain that there is one. Functional limitations are expected to continuously increase in the periods before and after a nursing home admission: the goal of an admission is not to improve a patient’s health or their functioning but instead help them to cope with these limitations. The identification strategy makes individuals comparable in physical health in the six-month period before nursing home admission, and hence the deterioration in physical limitations should still be observable in our estimates. This helped to assess whether the observed halt in well-being scores after admission is not the result of our identification strategy unintentionally removing differences in functional limitations between cohorts.

**References:**

CBS. (2015). *Gezondheidsmonitor 2012*. Opbouw en instructie totaalbestand. https://www.cbs.nl/nl-nl/onze-diensten/maatwerk-en-microdata/microdata-zelf-onderzoek-doen/microdatabestanden/gezondheidsmonitor-2012-opbouw-en-instructie-totaalbestand

CBS. (2017). Documentatie Gezondheidsmonitor Volwassen en Ouderen 2016. https://www.volksgezondheidenzorg.info/sites/default/files/gemon.pdf

de Jong Gierveld, J., & van Tilburg, T. G. (1999). *Manual of the loneliness scale*. VU University Amsterdam, Department of Social Research

Methodology.

1. Listening to a conversation with 3 or more persons; having a conversation with one person; reading the small print of a newspaper; recognizing someone’s face at a distance of 4 meters; carrying an item of 5kg for 10 meters; reaching for something on the ground; walking for 400 meters without standing still. [↑](#footnote-ref-1)
